# Utility of single versus sequential measurements of risk factors for prediction of stroke in Chinese adults

**DOI:** 10.1038/s41598-021-95244-8

**Published:** 2021-09-02

**Authors:** Matthew Chun, Robert Clarke, Tingting Zhu, David Clifton, Derrick Bennett, Yiping Chen, Yu Guo, Pei Pei, Jun Lv, Canqing Yu, Ling Yang, Liming Li, Zhengming Chen, Benjamin J. Cairns, the China Kadoorie Biobank Collaborative Group, Junshi Chen, Zhengming Chen, Robert Clarke, Rory Collins, Yu Guo, Liming Li, Jun Lv, Richard Peto, Robin Walters, Daniel Avery, Derrick Bennett, Ruth Boxall, Fiona Bragg, Sushila Burgess, Kahung Chan, Yumei Chang, Yiping Chen, Zhengming Chen, Robert Clarke, Huaidong Du, Zammy Fairhurst-Hunter, Simon Gilbert, Alex Hacker, Parisa Hariri, Michael Holmes, Andri Iona, Becky Im, Maria Kakkoura, Christiana Kartsonaki, Rene Kerosi, Kuang Lin, Iona Millwood, Qunhua Nie, Alfred Pozaricki, Paul Ryder, Sam Sansome, Dan Schmidt, Rajani Sohoni, Rebecca Stevens, Iain Turnbull, Robin Walters, Lin Wang, Neil Wright, Ling Yang, Xiaoming Yang, Pang Yao, Yu Guo, Xiao Han, Can Hou, Chao Liu, Jun Lv, Pei Pei, Canqing Yu, Chun Li, Zengchang Pang, Ruqin Gao, Shanpeng Li, Shaojie Wang, Yongmei Liu, Ranran Du, Liang Cheng, Xiaocao Tian, Hua Zhang, Yaoming Zhai, Feng Ning, Xiaohui Sun, Feifei Li, Silu Lv, Junzheng Wang, Wei Hou, Mingyuan Zou, Shichun Yan, Xue Zhou, Bo Yu, Yanjie Li, Qinai Xu, Quan Kang, Ziyan Guo, Ximin Hu, Jinyan Chen, Xiaohuan Wang, Min Weng, Zhendong Guo, Shukuan Wu, Yilei Li, Huimei Li, Ming Wu, Yonglin Zhou, Jinyi Zhou, Ran Tao, Jie Yang, Jian Su, Fang Liu, Jun Zhang, Yihe Hu, Yan Lu, Liangcai Ma, Aiyu Tang, Yujie Hua, Jianrong Jin, Jingchao Liu, Zhenzhu Tang, Naying Chen, Duo Liu, Mingqiang Li, Jinhuai Meng, Rong Pan, Qilian Jiang, Jian Lan, Yun Liu, Liuping Wei, Liyuan Zhou, Ningyu Chen, Ping Wang, Fanwen Meng, Yulu Qin, Sisi Wang, Xianping Wu, Ningmei Zhang, Xiaofang Chen, Xunfu Zhong, Jiaqiu Liu, Qiang Sun, Guojin Luo, Jianguo Li, Xiaofang Chen, Xunfu Zhong, Jiaqiu Liu, Qiang Sun, Pengfei Ge, Xiaolan Ren, Caixia Dong, Hui Zhang, Enke Mao, Zhongxiao Li, Tao Wang, Xi Zhang, Ding Zhang, Gang Zhou, Shixian Feng, Liang Chang, Lei Fan, Yulian Gao, Tianyou He, Huarong Sun, Pan He, Chen Hu, Xukui Zhang, Min Yu, Ruying Hu, Hao Wang, Weiwei Gong, Meng Wang, Chunmei Wang, Xiaoyi Zhang, Kaixu Xie, Lingli Chen, Dongxia Pan, Qijun Gu, Yuelong Huang, Biyun Chen, Li Yin, Huilin Liu, Zhongxi Fu, Qiaohua Xu, Xin Xu, Hao Zhang, Huajun Long, Libo Zhang

**Affiliations:** 1 Clinical Trial Service Unit and Epidemiological Studies, Nuffield Department of Population Health, University of Oxford, Big Data Institute, Old Road Campus, Oxford, OX 7LF UK; 2 Department of Engineering Science, University of Oxford, Oxford, UK; 3 Oxford-Suzhou Centre for Advanced Research, Suzhou, China; 4 Medical Research Council, Population Health Research Unit, University of Oxford, Oxford, UK; 5 Chinese Academy of Medical Sciences, Beijing, China; 6 Department of Epidemiology and Biostatistics, School of Public Health, Peking University Health Sciences Center, Beijing, China; 7 Peking University Center for Public Health and Epidemic Preparedness & Response, Beijing, China; 8 China National Center For Food Safety Risk Assessment, Beijing, China; 9 Qingdao CDC, Qingdao, Shangdong China; 10 Licang CDC, Licang District, Qingdao, Shandong China; 11 Heilongjiang CDC, Harbin, Heilongjiang, China; 12 Nangang CDC, Nangang District, Harbin, Heilongjiang, China; 13 Hainan CDC, Haikou, Hainan China; 14 Meilan CDC, Meilang District, Haikou, Hainan China; 15 Jiangsu CDC, Nanjing, Jiangsu China; 16 Suzhou CDC, Suzhou, Jiangsu China; 17 Guangxi CDC, Nanning, Guangxi China; 18 Liuzhou CDC, Liuzhou, Guangxi China; 19 Sichuan CDC, Chengdu, Sichuan China; 20 Pengzhou CDC, Pengzhou, Sichuan China; 21 Gansu CDC, Lanzhou, Gansu China; 22 Maiji CDC, Maiji, Tianshui, Gansu China; 23 Henan CDC, Zhengzhou, Henan China; 24 Huixian CDC, Huixian, Henan China; 25 Zhejiang CDC, Hanzhou Zhejiang, China; 26 Tongxiang CDC, Tongxiang, Zhejiang China; 27 Hunan CDC, Changsha, Hunan China; 28 Liuyang CDC, Liuyang, Hunan China

**Keywords:** Neuroscience, Health care, Neurology, Risk factors

## Abstract

Absolute risks of stroke are typically estimated using measurements of cardiovascular disease risk factors recorded at a single visit. However, the comparative utility of single versus sequential risk factor measurements for stroke prediction is unclear. Risk factors were recorded on three separate visits on 13,753 individuals in the prospective China Kadoorie Biobank. All participants were stroke-free at baseline (2004–2008), first resurvey (2008), and second resurvey (2013–2014), and were followed-up for incident cases of first stroke in the 3 years following the second resurvey. To reflect the models currently used in clinical practice, sex-specific Cox models were developed to estimate 3-year risks of stroke using single measurements recorded at second resurvey and were retrospectively applied to risk factor data from previous visits. Temporal trends in the Cox-generated risk estimates from 2004 to 2014 were analyzed using linear mixed effects models. To assess the value of more flexible machine learning approaches and the incorporation of longitudinal data, we developed gradient boosted tree (GBT) models for 3-year prediction of stroke using both single measurements and sequential measurements of risk factor inputs. Overall, Cox-generated estimates for 3-year stroke risk increased by 0.3% per annum in men and 0.2% per annum in women, but varied substantially between individuals. The risk estimates at second resurvey were highly correlated with the annual increase of risk for each individual (men: r = 0.91, women: r = 0.89), and performance of the longitudinal GBT models was comparable with both Cox and GBT models that considered measurements from only a single visit (AUCs: 0.779–0.811 in men, 0.724–0.756 in women). These results provide support for current clinical guidelines, which recommend using risk factor measurements recorded at a single visit for stroke prediction.

## Introduction

Stroke is a leading cause of death and disability worldwide, and China accounts for approximately one-third of the global burden of stroke deaths^1^. Current guidelines for primary prevention of stroke advocate use of risk prediction equations, including the Pooled Cohort Equations^2^ for cardiovascular disease (CVD) or the Framingham Stroke Risk Profile^3^ for stroke^4,5^. Such risk scores are typically derived using Cox proportional hazards models that generate risk predictions based on measurements of risk factors recorded at a single baseline visit. However, adults without established CVD are recommended to have their risk reassessed every 3–5 years^6–8^, and it remains unclear if CVD prediction can be improved using repeated measurements of time-dependent risk factors including blood pressure (BP), cholesterol levels, body weight, and lifestyle factors. BP has been extensively studied, and while longitudinal indices of BP appear to have incrementally stronger associations with CVD than “present” BP, head-to-head comparisons of their predictive utility for incident disease are limited^9^. Previous studies comparing the predictive utility of sequential versus single risk factor measurements have reported conflicting results, ranging from no improvement^10,11^ to statistically significant incremental improvements (ΔC-index: 0.0023–0.0040)^12^ or substantial improvements (ΔC-index: 0.05–0.06)^13,14^, despite using similar modeling approaches. Moreover, none of these previous studies examined major risk factors for incident stroke or involved the Chinese population, in which incidence of stroke is particularly high.

Consequently, substantial uncertainty persists about the relevance of sequential measurements of established CVD risk factors recorded on two or three separate visits versus those recorded on a single visit for prediction of incident stroke in Chinese adults. The aims of the present study were: (i) to examine changes in CVD risk factors and 3-year absolute risks of stroke in Chinese adults from 2004–2014, and (ii) to compare the utility of sequential versus single measurements of CVD risk factors for prediction of stroke in a contemporary study of Chinese adults.

## Methods

### Data sources

The China Kadoorie Biobank (CKB)^15,16^ is a prospective cohort study of 512,726 adults who were enrolled from 10 geographically-diverse areas (5 urban, 5 rural) of China in 2004–2008. In each area, all permanent residents without disability aged 35–74 years were invited to participate. An interviewer-administered electronic questionnaire was used to collect baseline risk factor input data including sociodemographic factors, lifestyle factors (e.g., smoking, alcohol, dietary habits), medical history and current medication, and physical activity. Physical measurements were also recorded, including height, weight, hip and waist circumference, bio-impedance, blood pressure, and heart rate. All participants provided a blood sample, and random blood glucose tests were conducted to screen for diabetes. Two resurveys were conducted in 2008 and 2013–2014, respectively, each including about 5% of the surviving CKB participants and recording information on the same risk factor inputs as were collected during the baseline survey (2004–2008). For the purposes of this study, we use the terms “risk factors” and “risk factor inputs” to refer to all of the CKB variables available to our models for risk prediction of stroke, regardless of whether they are considered established risk factors in clinical practice. Details of the variables assessed in the present report are provided in Supplementary Table 1.

All follow-up data on incident disease and cause-specific mortality outcomes were collected by linkage to death registries, established registries of major diseases, and health insurance records (covering > 97% of participants); local residential records; and annual home visits for uninsured participants through January 1, 2018. All stroke cases were coded by trained medical staff using the International Classification of Diseases 10th revision (ICD-10) (Supplementary Methods 1). Ethical approval for CKB was obtained from the Oxford University Tropical Research Ethics Committee and the Chinese Center for Disease Control and Prevention Ethical Review Committee, and all participants provided written informed consent.

The present analyses were restricted to 13,753 CKB participants (5,152 men; 8,601 women) who had three sequential measurements of risk factor inputs recorded at baseline survey, first resurvey, and second resurvey, and who had no prior history of stroke through the date of their second resurvey. Among these individuals, 644 incident cases of first-ever stroke (267 in men; 377 in women) were recorded within the first three years following the second resurvey.

### Statistical analysis

Included CKB individuals were randomly divided into a training set (85%) and test set (15%). The training set was used for model development while the test set was used for independent evaluation of model performance in order to avoid over-optimism. Risk factor data were preprocessed to maintain consistency between the variables recorded at each visit, and missing values (< 0.03% of all data) were imputed using sex-specific mean imputation (Supplementary Methods 2).

To reflect the Cox models currently used in clinical practice, such as the Pooled Cohort Equations^2^ and the Framingham Stroke Risk Profile^3^, we first developed regularized and region-stratified, sex-specific Cox models for 3-year risk prediction of stroke using data on risk factors recorded at the second resurvey. These Cox models utilize risk factors measurements recorded at a single visit for prediction of future stroke events and serve as an established gold standard against which other modeling approaches can be compared. Further details of the Cox model development process are provided in Supplementary Methods 3, with the final set of included risk factors outlined in Supplementary Table 1.

In order to analyze temporal trends in absolute risks of stroke among Chinese individuals from 2004 to 2014, the Cox models (developed based on second resurvey data only) were retrospectively applied, for each individual, to risk factor measurements also recorded at the baseline and first resurvey. This yielded a total of three separate risk estimates for each individual, with each estimate based on risk factor measurements recorded at a single visit. Population-level and individual-level trends in the risk estimates were analyzed using linear mixed effects models (LMEs) (Supplementary Methods 3).

Finally, to assess the value of machine learning (ML) approaches and sequential measurements of risk factor inputs for 3-year prediction of stroke, we developed sex-specific gradient boosted tree (GBT) models using four modeling approaches and compared their performance against the Cox models. GBT was chosen because of its ability to model non-linear interactions between risk factor inputs, incorporate longitudinal features, and achieve best-in-class performance for stroke risk prediction in other recent studies^14,17^.

In the first GBT modeling approach, we used only single measurements of risk factor inputs recorded at the second resurvey in order to provide a direct comparison against the Cox models, which were developed using the same input data. In the second GBT modeling approach, we used the raw sequential measurements of risk factor inputs recorded at all three visits (i.e., baseline, first resurvey, second resurvey) with no further feature engineering. In the third GBT modeling approach, we engineered features to provide a longitudinal summary of the sequential measurements recorded at all three visits, in a similar manner reported by Cho et al.^13^ and Zhao et al.^14^. Namely, we provided the models with (i) mean, standard deviation, minimum, and maximum values recorded for continuous risk factor inputs, and (ii) mean and standard deviation of recorded values for binary risk factor inputs. In the fourth GBT modeling approach, we engineered features to provide a longitudinal summary of the Cox-generated risk estimates at all three visits and combined these with single measurements of risk factor inputs recorded at the second resurvey. This fourth modeling approach was adapted from two-stage modeling approach described by Sweeting et al.^10^, and the engineered features included (i) individual-level random slopes and random intercepts from the linear mixed effects models, (ii) variance of Cox-generated risk estimates across the three visits, and (iii) absolute changes in Cox-generated risk estimates between consecutive visits. While the third modeling approach incorporated longitudinal summaries of the individual risk factor inputs, the fourth modeling approach provided a longitudinal summary of the overall stroke risk estimates for each individual. All GBT hyperparameters were tuned to maximize AUC using repeated threefold cross-validated grid searches within the training set, and all GBT models were trained and calibrated with isotonic regression using threefold cross-validation. Additional details of the GBT model development process are provided in Supplementary Methods 3.

All models were evaluated for predictive performance using the test set, in which we compared the performance of the Cox and GBT models for predicting stroke during the 3 years following the second resurvey. Model discrimination and calibration were assessed using area under the receiver operating characteristic curve (AUC) and χ^2^ statistics from the Hosmer–Lemeshow (for binary classification models) and Nam-D’Agostino (for survival models) tests. Confidence intervals for AUCs were constructed using 1,000 bootstrapped samples from the test set. Better risk discrimination was indicated by higher AUCs, and better calibration was indicated by lower χ^2^ values. To assess the changes in predictive performance of each modeling approach against the Cox models, we performed Delong’s test^18^ for ΔAUCs and calculated category-free net reclassification index (NRI) and integrated discrimination improvement (IDI). Statistical analyses were performed using Python version 3.7.0 and R version 3.6.1.

## Results

Among the 13,753 CKB participants (5,152 men; 8,601 women), 644 incident cases of first-ever stroke (267 in men; 377 in women) were recorded within the first three years following the second resurvey. Compared with those who had no stroke, individuals who had a first stroke were older and more likely to have prior history of coronary heart disease (CHD), diabetes, or hypertension (Table 1). In both men and women, age, prior history of coronary heart disease, diabetes, use of antihypertensive medication, and mean levels of systolic blood pressure increased from baseline to second resurvey, while the prevalence of current smoking decreased. Additional details about the timing of risk factor measurements are provided in Supplementary Fig. 1. Likewise, further details about the time-evolution in distributions of well-established stroke risk factors are provided in Supplementary Figs. 2–7.

**Table 1:** Distribution of major risk factors for stroke in individuals at baseline (2004–2008), 1st resurvey (2008), and 2nd resurvey (2013–2014) in the China Kadoorie Biobank.

Men						
Risk factor	Baseline (2004–2008)		Resurvey 1 (2008)		Resurvey 2 (2013–2014)	
	No stroke (n = 4,885)	Total stroke (n = 267)	No stroke (n = 4,885)	Total stroke (n = 267)	No stroke (n = 4,885)	Total stroke (n = 267)
Age, mean, yr	51.3	60.3	53.9	62.9	59.3	68.4
Current smoking, %	68.7	61.4	65.8	58.1	55.9	50.4
Coronary heart disease, %	1.8	7.9	2.8	9.7	5.9	13.9
Age 65 yrs + , %	11.5	33.7	16.2	46.4	29.3	64.4
Diabetes, %	3.5	7.1	5.6	13.1	9.3	16.9
BP-lowering treatment, %	6.6	12.7	9.7	21.3	18.9	27.3
SBP, mean, mmHg	132	140	126	134	135	147

Women						
Risk factor	Baseline (2004–2008)		Resurvey 1 (2008)		Resurvey 2 (2013–2014)	
	No stroke (n = 8,224)	Total stroke (n = 377)	No stroke (n = 8,224)	Total stroke (n = 377)	No stroke (n = 8,224)	Total stroke (n = 377)
Age, mean, yr	50.1	57.1	52.7	59.8	58.2	65.3
Current smoking, %	2.9	4.2	2.5	3.2	2.2	2.4
Coronary heart disease, %	2.3	6.1	3.4	9.5	6.7	17.2
Age 65 yrs + , %	7.9	23.6	12.0	30.2	24.1	50.9
Diabetes, %	4.1	10.3	7.0	16.2	10.8	20.7
BP-lowering treatment, %	8.8	15.4	11.6	20.4	20.7	32.6
SBP, mean, mmHg	130	140	124	133	135	147

Note: “No Stroke” columns include individuals who remained stroke-free until being censored, even if lost to follow up before 3 years.

**Figure 1:**
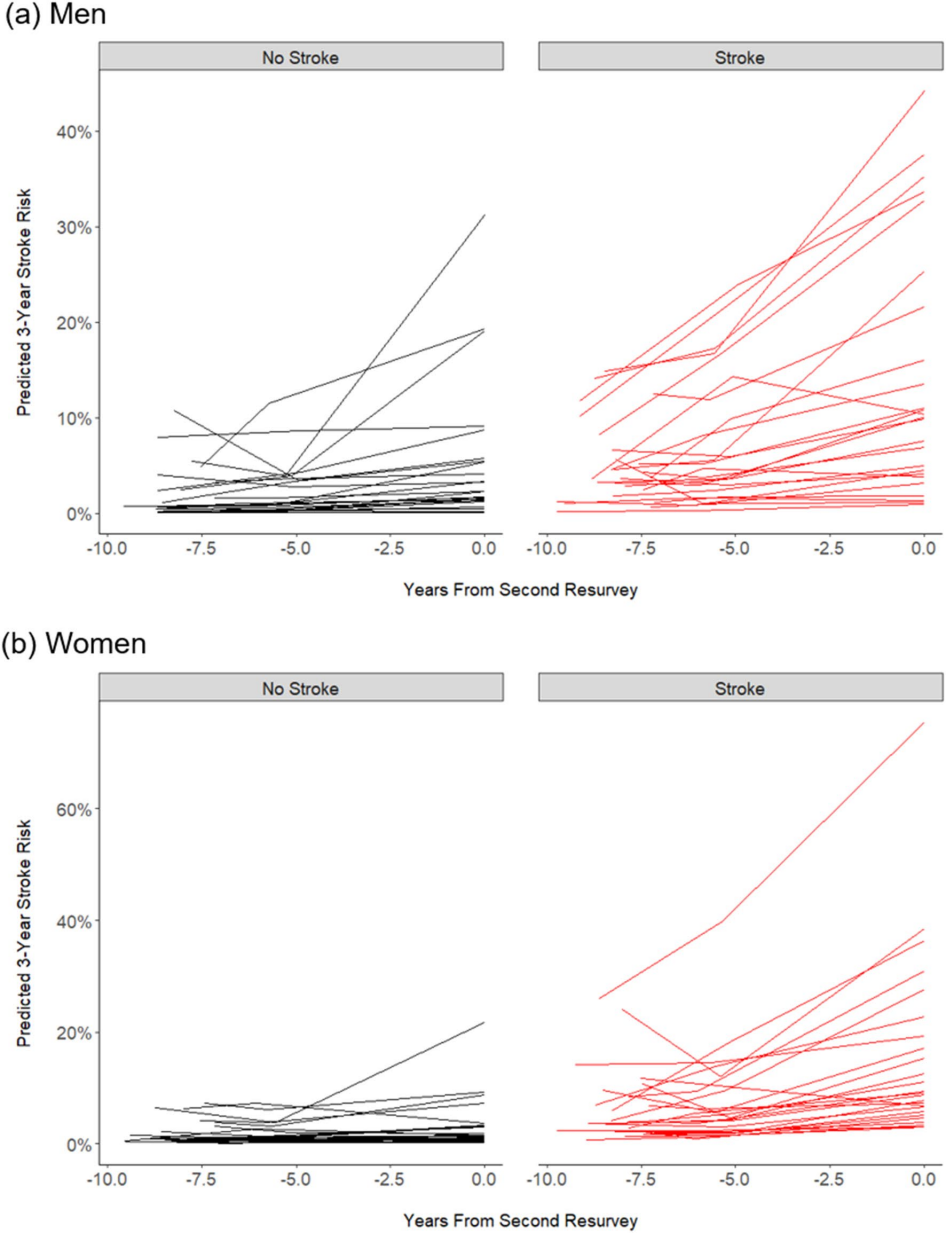
Temporal trends in predicted 3-year stroke risk for a random sample of (**a**) 50 men and (**b**) 50 women from the China Kadoorie Biobank, stratified by incident stroke outcomes.

**Figure 2:**
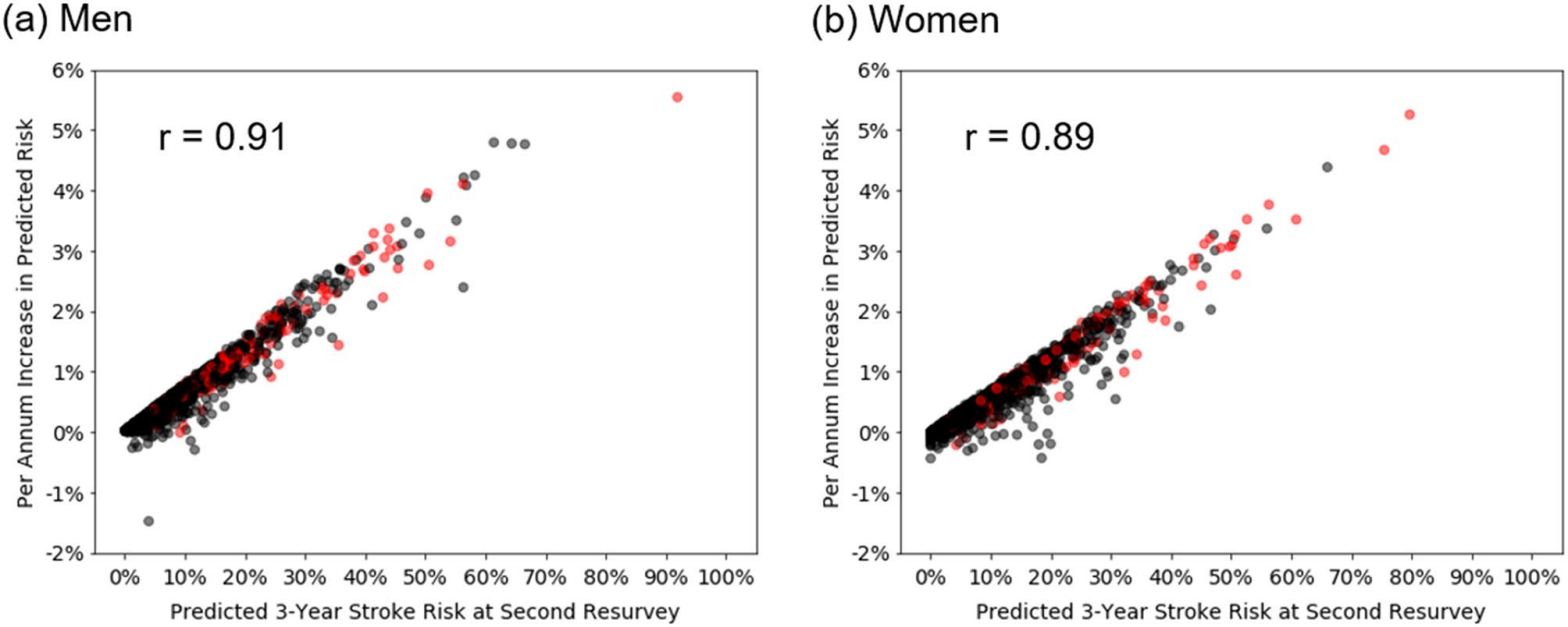
Relationship between predicted 3-year stroke risk at second resurvey and per annum increase in predicted risk (fixed effect slope + random effect slope from linear mixed effects model) for (**a**) men and (**b**) women in the China Kadoorie Biobank. Black points: individuals with no stroke; Red points: individuals with stroke.

According to the Cox models, these temporal risk factor trends yielded increasing 3-year predicted stroke risks separately in individuals with and without stroke (Fig. 1). On average, 3-year predicted stroke risks increased by 0.3% per annum in men and 0.2% per annum in women, but these risks differed substantially between individuals (Fig. 1). Among those with stroke, predicted risk increased 1.0% per annum in men and 0.8% per annum in women, with average predicted risk at second resurvey being 13.9% in men and 12.0% in women. In contrast, among those without stroke, predicted risk increased only 0.3% per annum in men and 0.2% per annum in women, with average predicted risk at second resurvey being 4.3% in men and 3.7% in women. In both sexes, the predicted stroke risk at second resurvey was highly correlated with the annual increase of risk for each individual over the previous decade (men: r = 0.91, women: r = 0.89; Fig. 2).

The Cox models, which estimated risk of stroke based on a single measurement of risk factors at second resurvey, predicted incident stroke in the subsequent three years with good discrimination (AUC: 0.779 in men, 0.756 in women) and calibration (χ^2^: 16.8 in men, 17.3 in women) (Table 2). Overall, the GBT models yielded slightly higher AUCs in men (0.795–0.811) and lower AUCs in women (0.724–0.750) than the Cox models (Table 2). However, none of these differences were statistically significant (Table 3) and there was substantial variability across bootstrapped samples. Except for the fourth GBT modeling approach (longitudinal summary of stroke risk estimates at three visits + single measurement of risk factor inputs at most recent visit), the GBT models generally appeared to be better calibrated than the Cox models in both men and women (Table 2; Supplementary Figs. 8–12). The NRI and IDI values were negative for almost all GBT models. However, once again, the confidence intervals of these values typically included zero (no difference), indicating no significant change in their predictive utility.

**Table 2:** Comparison of discrimination and calibration performance for prediction of 3-year risk of stroke using Cox and Gradient Boosted Tree (GBT) modeling approaches.

Modeling Approach and Included Data	Men		Women	
	Discrimination AUC [95%CI]	Calibration χ^2^ (p-value)	Discrimination AUC [95%CI]	Calibration χ^2^ (p-value)
**Single measurement of risk factor inputs**				
Cox: Single measurement at most recent visit	0.779 [0.709–0.845]	16.8 (p = 0.05)	0.756 [0.692–0.814]	17.3 (p = 0.04)
GBT: Single measurement at most recent visit	0.811 [0.753–0.867]	5.6 (p = 0.78)	0.743 [0.681–0.798]	7.3 (p = 0.61)
**Sequential measurements of risk factor inputs**				
GBT: Sequential measurements at three visits	0.795 [0.721–0.858]	1.9 (p = 0.99)	0.741 [0.677–0.796]	14.3 (p = 0.11)
GBT: Longitudinal summary ^a^ of sequential measurements at three visits	0.789 [0.721–0.851]	5.0 (p = 0.83)	0.724 [0.660–0.782]	9.2 (p = 0.42)
GBT: Longitudinal summary of stroke risk estimates ^b^ at three visits + Single measurement at most recent visit	0.786 [0.719–0.851]	29.5 (p < 0.01)	0.750 [0.683–0.811]	20.2 (p = 0.02)

^a^ Longitudinal summary of risk factor inputs included (i) mean, standard deviation, minimum, and maximum values recorded for continuous risk factor inputs, and (ii) mean and standard deviation for binary risk factor inputs.

^b^ Longitudinal summary of risk estimates included individual-level random slopes and random intercepts from linear mixed effects models; variance of Cox-generated risk estimates across three visits; and absolute changes in Cox-generated risk estimates between consecutive visits.

**Table 3:** Net reclassification index (NRI) and integrated discrimination index (IDI) of Cox and Gradient Boosted Tree (GBT) modeling approaches for predicting 3-year risk of stroke.

Modeling approach and included data	Men			Women		
	ΔAUC (p-value)	NRI [95%CI]	IDI [95%CI]	ΔAUC (p-value)	NRI [95%CI]	IDI [95%CI]
**Single measurement of risk factor inputs**						
Cox: Single measurement at most recent visit	Referent	Referent	Referent	Referent	Referent	Referent
GBT: Single measurement at most recent visit	+ 0.032 (p = 0.08)	+ 0.14 [−0.17, 0.44]	−0.03 [−0.08, 0.01]	−0.013 (p = 0.59)	−0.25 [−0.51, 0.01]	−0.03 [−0.06, −0.01]
**Sequential measurements of risk factor inputs**						
GBT: Sequential measurements at three visits	+ 0.016 (p = 0.57)	−0.20 [−0.52, 0.12]	−0.03 [−0.09, 0.02]	−0.015 (p = 0.42)	−0.13 [−0.39, 0.14]	−0.03 [−0.06, −0.01]
GBT: Longitudinal summary ^a^ of sequential measurements at three visits	+ 0.010 (p = 0.77)	−0.13 [−0.45, 0.18]	−0.05 [−0.11, 0.01]	−0.032 (p = 0.14)	−0.29 [−0.55, −0.03]	−0.04 [−0.06, −0.01]
GBT: Longitudinal summary of stroke risk estimates ^b^ at three visits + Single measurement at most recent visit	+ 0.007 (p = 0.38)	−0.02 [−0.34, 0.30]	0.00 [−0.02, 0.02]	−0.006 (p = 0.32)	+ 0.15 [−0.11, 0.42]	0.00 [−0.01, 0.02]

^a^ Longitudinal summary of risk factor inputs included (i) mean, standard deviation, minimum, and maximum values recorded for continuous risk factor inputs, and (ii) mean and standard deviation for binary risk factor inputs.

^b^ Longitudinal summary of risk estimates included individual-level random slopes and random intercepts from linear mixed effects models; variance of Cox-generated risk estimates across three visits; and absolute changes in Cox-generated risk estimates between consecutive visits.

Rankings of the top-10 most important features with corresponding Gini importance scores for all GBT models are provided in Supplementary Tables 2–3. Across all models, age, blood pressure variables, measurements of size and weight, exercise, and region were identified as important features. However, in the fourth GBT modeling approach, in which a longitudinal summary of the Cox-risk estimates were provided as inputs, these longitudinal features had higher Gini importance scores than any individual risk factor inputs across all of the GBT models.

## Discussion

The present report highlighted temporal changes in mean levels of major stroke risk factors and 3-year absolute risks of stroke in 13,753 geographically-diverse Chinese adults, including 644 incident cases of first stroke occurring between 2004 and 2014. Among men and women who were stroke-free until the second resurvey (2013–2014), the predicted absolute risks of stroke throughout the previous decade increased with each consecutive year of follow-up, together with increasing numbers of individuals with coronary heart disease and diabetes, and increased use of blood pressure-lowering medication. Despite wide variations between individuals, the annual rate of increase of predicted stroke risk was about 3 to fourfold greater for individuals with stroke compared with those without stroke in the 3 years following the second resurvey. These results suggest that temporal trends in stroke risk can be informative for prediction of incident stroke events.

Nevertheless, while temporal changes in risk are informative for prediction of stroke, the sequential measurements provided limited additional value for prediction compared with risk factors recorded at a single visit. Because of the high correlation between an individual’s predicted risk at second resurvey and their per annum increase in risk, the predicted risk at the most recent visit was informative of an individual’s temporal risk profile prior to that visit. The individuals at highest risk for stroke at the second resurvey (2013–2014) were largely the same individuals with the fastest growing risk throughout the previous decade. This correlation is an established phenomenon in epidemiology referred to as the “horse-racing effect”^19^, by analogy to the fact that horses leading a race at any given moment are those which have been running the fastest up to that point. Thus, the incorporation of temporal information from sequential risk factor measurements did not yield any improved risk prediction compared to models developed using single measurements of risk factor inputs. The results of the present study confirm the utility of single rather than sequential measurements for risk prediction of stroke, as currently recommended in clinical practice^4,5^.

Other studies^10–14^ have investigated the utility of sequential risk factor measurements for improving prediction of CVD, but they have not focused specifically on prediction of stroke or risk assessment in a Chinese population. Consistent with the findings of the present study, comparable analyses of approximately 13,000 individuals from the Atherosclerosis Risk in Communities (ARIC) study reported no improvements in AUC/C-index for 3-year risk prediction of CVD^10^ (albeit with large standard errors due to a small number of events), or for prediction of coronary heart disease^11^, despite exploring a variety of modeling approaches. A larger study involving almost 200,000 participants from the Emerging Risk Factors Collaboration explored the inclusion of repeated risk factor measurements for 5-year CVD prediction, and reported statistically significant but marginal improvements of C-index ranging from 0.0023 to 0.0040^12^. Meanwhile, a study of 0.5 M Korean adults found more substantial improvements in CVD prediction (ΔC-index: 0.05–0.06) by including a longitudinal summary of sequential risk factor measurements recorded at periodic health screenings^13^, and a study using electronic health records data of 109,490 individuals from the Vanderbilt University Medical Center reported improvements in AUC of 0.05–0.06 for 10-year CVD prediction using GBT models with longitudinal inputs.

The range of findings between these studies can potentially be attributed to differences between study settings, risk factors included, and the frequency or quantity of repeated risk factor measurements. It has been suggested that predictive improvement, if any, from sequential risk factor measurements may reflect the ability to account for measurement error and variability in risk factors, with no substantial further benefit derived from estimating individual-level slopes of risk factors over time^10,12,13^. Consequently, it is not surprising that the predictive benefit of longitudinal risk factor data can vary greatly depending on the particular characteristics of a study and the underlying cohort. While the present study had appropriate statistical power to detect substantial improvements in AUC of 0.05–0.06 reported by Cho et al.^13^ and Zhao et al.^14^, greater statistical power would be required to detect the much more modest improvements of less than 0.01 reported by Paige et al.^12^. Since AUC is well-known to be insensitive for detecting small differences in discriminative ability between models^20^, we have also characterized the models in this study using NRI and IDI. The validity of using these metrics has been criticized for their risk of false positive indications of predictive differences between models^21^. However, even for these overly sensitive metrics, there was little or no evidence for differences in predictive performance between each modeling approach.

The limitations of the present study include (i) lack of data on certain established risk factors for stroke such as atrial fibrillation (AF) and blood cholesterol levels; (ii) quality of self-reported questionnaire data about medical history of coronary heart disease or diabetes; and (iii) inclusion of only three sequential measurements of risk factors recorded over 10 years. Importantly, a recent population-based study of Chinese adults involving comparable age groups^22^, reported that the prevalence of AF is low in China (0.71% in adults aged 35 years or older). Consequently, although AF is an important predictor of stroke, its omission is likely to have a material effect for only a small proportion of individuals in the present study. Similarly, mean blood levels of total cholesterol and LDL cholesterol are well-known to be lower in China than in Western populations and may be less important for stroke risk prediction in this population^23^. To adjust for errors in self-reported prior medical history, we considered an individual to have a history of coronary heart disease or diabetes if they were ever reported or detected (e.g., by blood glucose screening) to have such disease on any previous visit. Finally, while the present study included only three sequential measurements of risk factors recorded over 10 years, increased number or frequency of measurements may still be useful for improving stroke prediction and should be investigated further.

## Conclusions

Individuals at highest risk for stroke in China from 2013 to 2014 were largely the same individuals who had the fastest growing risk of stroke throughout the previous decade. However, in this population, measurements of CVD risk factors for prediction of stroke at three separate surveys had limited additional utility compared to those recorded at a single survey. These results provide further support for current guidelines for clinical practice, which suggest that stroke prediction is best estimated and cost-effectively implemented using models involving measurements of major risk factors recorded at a single visit.

### Ethics approval

Ethical approval for CKB was obtained from the Oxford University Tropical Research Ethics Committee and the Chinese Center for Disease Control and Prevention Ethical Review Committee.

### Consent to participate

All participants provided written informed consent.

### Consent for publication

All authors approved the final version of this report.

## Supplementary Information

Supplementary Information.

## Data Availability

The data supporting analyses included in the present study are available from the corresponding authors upon reasonable request.

## Code availability

Code supporting this study are available from the corresponding authors upon reasonable request.
