# Publisher Correction: Utility of single versus sequential measurements of risk factors for prediction of stroke in Chinese adults

**DOI:** 10.1038/s41598-021-00401-8

**Published:** 2021-10-18

**Authors:** Matthew Chun, Robert Clarke, Tingting Zhu, David Clifton, Derrick Bennett, Yiping Chen, Yu Guo, Pei Pei, Jun Lv, Canqing Yu, Ling Yang, Liming Li, Zhengming Chen, Benjamin J. Cairns, the China Kadoorie Biobank Collaborative Group, Junshi Chen, Zhengming Chen, Robert Clarke, Rory Collins, Yu Guo, Liming Li, Jun Lv, Richard Peto, Robin Walters, Daniel Avery, Derrick Bennett, Ruth Boxall, Fiona Bragg, Sushila Burgess, Kahung Chan, Yumei Chang, Yiping Chen, Zhengming Chen, Robert Clarke, Huaidong Du, Zammy Fairhurst-Hunter, Simon Gilbert, Alex Hacker, Parisa Hariri, Michael Holmes, Andri Iona, Becky Im, Maria Kakkoura, Christiana Kartsonaki, Rene Kerosi, Kuang Lin, Iona Millwood, Qunhua Nie, Alfred Pozaricki, Paul Ryder, Sam Sansome, Dan Schmidt, Rajani Sohoni, Rebecca Stevens, Iain Turnbull, Robin Walters, Lin Wang, Neil Wright, Ling Yang, Xiaoming Yang, Pang Yao, Yu Guo, Xiao Han, Can Hou, Chao Liu, Jun Lv, Pei Pei, Canqing Yu, Chun Li, Zengchang Pang, Ruqin Gao, Shanpeng Li, Shaojie Wang, Yongmei Liu, Ranran Du, Liang Cheng, Xiaocao Tian, Hua Zhang, Yaoming Zhai, Feng Ning, Xiaohui Sun, Feifei Li, Silu Lv, Junzheng Wang, Wei Hou, Mingyuan Zou, Shichun Yan, Xue Zhou, Bo Yu, Yanjie Li, Qinai Xu, Quan Kang, Ziyan Guo, Ximin Hu, Jinyan Chen, Xiaohuan Wang, Min Weng, Zhendong Guo, Shukuan Wu, Yilei Li, Huimei Li, Ming Wu, Yonglin Zhou, Jinyi Zhou, Ran Tao, Jie Yang, Jian Su, Fang Liu, Jun Zhang, Yihe Hu, Yan Lu, Liangcai Ma, Aiyu Tang, Yujie Hua, Jianrong Jin, Jingchao Liu, Zhenzhu Tang, Naying Chen, Duo Liu, Mingqiang Li, Jinhuai Meng, Rong Pan, Qilian Jiang, Jian Lan, Yun Liu, Liuping Wei, Liyuan Zhou, Ningyu Chen, Ping Wang, Fanwen Meng, Yulu Qin, Sisi Wang, Xianping Wu, Ningmei Zhang, Xiaofang Chen, Xunfu Zhong, Jiaqiu Liu, Qiang Sun, Guojin Luo, Jianguo Li, Xiaofang Chen, Xunfu Zhong, Jiaqiu Liu, Qiang Sun, Pengfei Ge, Xiaolan Ren, Caixia Dong, Hui Zhang, Enke Mao, Zhongxiao Li, Tao Wang, Xi Zhang, Ding Zhang, Gang Zhou, Shixian Feng, Liang Chang, Lei Fan, Yulian Gao, Tianyou He, Huarong Sun, Pan He, Chen Hu, Xukui Zhang, Min Yu, Ruying Hu, Hao Wang, Weiwei Gong, Meng Wang, Chunmei Wang, Xiaoyi Zhang, Kaixu Xie, Lingli Chen, Dongxia Pan, Qijun Gu, Yuelong Huang, Biyun Chen, Li Yin, Huilin Liu, Zhongxi Fu, Qiaohua Xu, Xin Xu, Hao Zhang, Huajun Long, Libo Zhang

**Affiliations:** 1 Clinical Trial Service Unit and Epidemiological Studies, Nuffield Department of Population Health, University of Oxford, Big Data Institute, Old Road Campus, Oxford, OX 7LF UK; 2 Department of Engineering Science, University of Oxford, Oxford, UK; 3 Oxford-Suzhou Centre for Advanced Research, Suzhou, China; 4 Medical Research Council, Population Health Research Unit, University of Oxford, Oxford, UK; 5 Chinese Academy of Medical Sciences, Beijing, China; 6 Department of Epidemiology and Biostatistics, School of Public Health, Peking University Health Sciences Center, Beijing, China; 7 Peking University Center for Public Health and Epidemic Preparedness & Response, Beijing, China; 8 China National Center For Food Safety Risk Assessment, Beijing, China; 9 Qingdao CDC, Qingdao, Shangdong China; 10 Licang CDC, Licang District, Qingdao, Shandong China; 11 Heilongjiang CDC, Harbin, Heilongjiang, China; 12 Nangang CDC, Nangang District, Harbin, Heilongjiang, China; 13 Hainan CDC, Haikou, Hainan China; 14 Meilan CDC, Meilang District, Haikou, Hainan China; 15 Jiangsu CDC, Nanjing, Jiangsu China; 16 Suzhou CDC, Suzhou, Jiangsu China; 17 Guangxi CDC, Nanning, Guangxi China; 18 Liuzhou CDC, Liuzhou, Guangxi China; 19 Sichuan CDC, Chengdu, Sichuan China; 20 Pengzhou CDC, Pengzhou, Sichuan China; 21 Gansu CDC, Lanzhou, Gansu China; 22 Maiji CDC, Maiji, Tianshui, Gansu China; 23 Henan CDC, Zhengzhou, Henan China; 24 Huixian CDC, Huixian, Henan China; 25 Zhejiang CDC, Hanzhou Zhejiang, China; 26 Tongxiang CDC, Tongxiang, Zhejiang China; 27 Hunan CDC, Changsha, Hunan China; 28 Liuyang CDC, Liuyang, Hunan China

Correction to: *Scientific Reports* 10.1038/s41598-021-95244-8, published online 02 September 2021

The original version of this Article contained an error in the spelling of author names Zhengming Chen and Liming Li, which were incorrectly given as Zhengming Chen (PI) and Liming Li (PI) in the China Kadoorie Biobank Collaborative Group.

The original Article has been corrected.

