# Supplementary material for: Utility of single versus sequential measurements of risk factors for prediction of stroke in Chinese adults
Source: Sci Rep. 2021 Sep 2;11:17575. doi: 10.1038/s41598-021-95244-8 (PMC8413314; doi:10.1038/s41598-021-95244-8)
Supplement: Supplementary file 1 — Supplementary Information. [file 41598_2021_95244_MOESM1_ESM.docx]

**Supplementary Materials**

**Supplementary Methods 1.** Follow up and coding for stroke cases

The vital status of participants was monitored through death registries supplemented by annual checks with local residential records and active confirmation by contacting local street committees or village administrators.^1^ All hospitalized cases of stroke were identified by electronic linkage to established registries of major diseases and health insurance records (covering >97% of participants), supplemented by annual home visits for uninsured participants.

All fatal and non-fatal stroke cases reported by different sources were coded using the International Classification of Diseases 10^th^ revision (ICD-10) by trained medical staff, who were blinded to other personal information, with further checking and review conducted centrally by trained medical staff. All hospital-reported cases of first stroke also underwent additional clinical adjudication, involving retrieval and review of original medical records and brain imaging reports by clinical specialists in China using a bespoke web-based system. About 92% of the reported first stroke cases had their diagnosis confirmed by brain imaging (CT or MRI). Radiological reports (but not primary brain images) of reported cases of non-fatal stroke were adjudicated by Chinese neurologists using a bespoke online system.^2^

**Supplementary Methods 2.** Data preprocessing

Data

Data used in this study were from CKB version 17.00.

Inclusion Criteria

In CKB, 14,999 individuals had data available from three separate visits at baseline, first resurvey, and second resurvey. Among these individuals, there were 1,760 incident strokes recorded before the end of follow up.

For some individuals included at second resurvey, only physical measurements were recorded without a full questionnaire administered. After removing these individuals (n=97), 14,902 individuals remained. Among these individuals, there were 1,733 incident strokes recorded before the end of follow up.

We excluded all individuals with stroke prior to second resurvey (either self-reported prior stroke or TIA at any visit, or follow-up data indicating a stroke event prior to the second resurvey). After removing these individuals (n=1,149), 13,753 individuals remained. Among these individuals, there were 835 incident strokes recorded before the end of follow up.

Finally, after considering only incident first stroke cases that occurred within 3 years from the second resurvey, we ended up with 13,753 included individuals (5,152 men; 8,601 women) with 644 first incident stroke cases (267 in men; 377 in women). The 3-year follow-up period was selected since some participants completed the second resurvey as late as 2014, with global end of follow-up being December 31, 2017.

Maintaining Consistency Between Surveys

In order to maintain consistency between risk factor inputs recorded at each visit, we considered only those that were recorded at all three surveys (i.e., baseline, first resurvey, and second resurvey). Region (including urban/rural classification) and sex were the only exceptions. Region and sex were recorded at the baseline survey and assumed to remain unchanged throughout the study.

Household income was recorded with additional high-income bands in the second resurvey, but for the purposes of this study, were standardized to match the wider income bands used in the baseline questionnaire and first resurvey.

Self-reported diagnoses of acute myocardial infarction, angina, and other ischemic heart disease were recorded separately at second resurvey, but were grouped together to match the variable for self-reported diagnoses of coronary heart disease at baseline and first resurvey.

Self-reported diagnoses of emphysema and bronchitis were recorded separately at second resurvey, but were grouped together to match the single variable for self-reported diagnoses of emphysema OR bronchitis at baseline and first resurvey.

Self-reported diagnoses of depression, anxiety, and other psychological disorders were recorded separately at second resurvey, but were grouped together to match the single variable for psychological disorders at baseline and first resurvey.

One-hot Encoding of Categorical Variables

For the Cox models, which were stratified by geographical area, categorical variables (excluding area), were one-hot encoded, resulting in a total of 398 binary risk factor inputs (“region” + “region_is_urban” + 132 risk factor inputs recorded at 3 visits). For all GBT models, geographical area was also one-hot encoded, resulting in 407 total risk factor inputs.

Training and Test Sets

CKB individuals were randomly divided into a training set (85%) and test set (15%) for model development and internal validation, respectively.

For survival models such as the Cox proportional hazards models developed in this study, right-censored individuals were included in both the training and test sets. The training set included 11,689 individuals (4,379 men; 7,310 women) with 547 incident first stroke cases during the 3-year follow-up period (227 in men; 320 in women). The test set included 2,064 individuals (773 men; 1,291 women) with 97 incident first stroke cases during the 3-year follow-up period (40 in men; 57 in women).

For binary prediction models such as the gradient-boosted tree models (GBTs) developed in this study, right-censored individuals who were lost to follow-up were excluded in both the training and test sets (since it is unknown whether or not they had a stroke during the full follow-up period). The training set included 11,464 individuals (4,259 men; 7,205 women) with 547 incident first stroke cases during the 3-year follow-up period (227 in men; 320 in women). The test set included 2,017 individuals (751 men; 1,266 women) with 97 incident first stroke cases during the 3-year follow-up period (40 in men; 57 in women).

Missing Values

Missing values were very rare in the CKB dataset, consisting of less than 0.03% of the risk factor input data. 318 of the 398 available risk factor input variables had no missing values.

Of the 80 risk factor input variables with missing values, there was a maximum of only 60 missing values for 79 of these features. Most of these variables corresponded to family medical history information. 10 individuals were also missing self-reported personal medical history information in the second resurvey. Fat percentage at second resurvey was the only outlier, having 166 missing values across the 13,753 included participants.

To address missing values for family medical history, 4 binary risk factor input variables were included to indicate missing medical history for the individual’s mother, father, siblings, or children, respectively, at each of the three surveys. All missing values were imputed using sex-specific mean values from the training set.

**Supplementary Methods 3.** Details of model development for Cox models, linear mixed effects models (LMEs), and gradient-boosted tree models (GBTs)

Cox Models:

Cox models were derived separately for men and women with stratification by China Kadoorie Biobank (CKB) area (5 urban and 5 rural regions). For each model, separate baseline survival functions were developed for each CKB area. The baseline hazard function (and corresponding baseline survival function) were derived from the non-parametric Breslow estimator.^3,4^ 10-fold cross-validated least absolute shrinkage and selection operator (LASSO) regularization was used within the training set for selecting a subset of risk factor inputs from all CKB variables. LASSO regularization was performed separately for each model, yielding slightly different numbers of selected risk factor inputs (66 risk factor inputs for men, 70 risk factor inputs for women). The specifics of the variable selection process have been previously described.^5,6^

After developing Cox models for predicting stroke in the 3 years following the second resurvey, the Cox models were retrospectively applied to risk factor input data recorded at baseline and first resurvey to assess 3-year predicted stroke risk at each visit.

Linear Mixed Effects Models (LMEs):

LMEs with individual-level random slopes and random intercepts were developed separately for each sex to analyze temporal trends in the Cox-generated 3-year stroke risk estimates for each individual. Intercepts were representative of predicted 3-year stroke risk at second resurvey while slopes were representative of annual changes in the 3-year predicted stroke risk in the decade preceding the second resurvey.

The form of the fitted LMEs was specified as:

$${Stroke\_Risk}_{ij}=\left( {b_{0j}+\beta}_{0} \right)+\left( {b_{1j}+\beta}_{1} \right)t_{ij}+\varepsilon_{ij}$$

$$b_{0j}\sim N(0,\tau_{1}^{2})$$

$$b_{1j}\sim N(0, \tau_{2}^{2})$$

$$cov{(b}_{0j},b_{1j})=\tau_{12}$$

where ${Stroke\_Risk}_{ij}$ denotes the 3-year stroke risk for visit *i* and individual *j*, $\beta_{0}$ represents the population-level fixed intercept, $\beta_{1}$ represents the population-level fixed slope, $b_{0j}$ represents the random intercept for individual *i*, $b_{1j}$ represents the random slope for individual *i*, $t_{ij}$ represents the time before second resurvey (in years), and $\varepsilon_{ij}$ represents the random error.

In order to make predictions for a previously unseen individual, a maximum likelihood estimate of the individual’s random slopes and random intercepts were determined based on the posterior distribution conditioned on the individual’s predicted risks and the trained LME parameters. These features, along with other longitudinal features and risk factor input data, were provided to GBTs for risk prediction, as described below.

LMEs were derived using the lmer() function of the lme4 package^7^ in R version 3.6.1.

Gradient Boosted Tree (GBT) Models:

GBT models were derived separately for both men and women using the GradientBoostingClassifier() function of the scikit-learn toolkit^8^ version 0.19.2. We trained the GBT models using four different modeling approaches, as described below:

1. GBT: Single measurement at most recent visit

These GBT models were trained using only single measurements of risk factor inputs recorded at the most recent visit (i.e., second resurvey).

1. GBT: Sequential measurements at three visits

These GBT models were trained using sequential measurements of risk factor inputs recorded at all three visits (i.e., baseline, first resurvey, second resurvey) with no further feature engineering.

1. GBT: Longitudinal summary of sequential measurements at three visits

These GBT models were trained with engineered features that provided a longitudinal summary of the sequential measurements recorded at all three visits. Namely, we provided the models with (i) mean, standard deviation, minimum, and maximum values recorded for continuous risk factor inputs, and (ii) mean and standard deviation values for binary risk factor inputs.

1. GBT: Longitudinal summary of stroke risk estimates at three visits + Single measurement at most recent visit

These GBT models were trained with engineered features that provided a longitudinal summary of the Cox-risk estimates at all three visits and combined these with the single, most-recent risk factor measurements recorded at the second resurvey.

*Hyperparameter Tuning for GBT Models*

All hyperparameters were tuned to maximize AUC in the validation set.

1. GBT: Single measurement at most recent visit

For men, learning_rate was initially fixed at 0.1, min_samples_split fixed at 2, min_samples_leaf fixed at 1, max_depth fixed at 8, max_features set to ‘sqrt’, and subsample fixed at 0.8. A first grid search was performed, with k-fold cross validation in the training set (k=3 folds selected based on runtime considerations), to tune n_estimators with values ranging from 5 to 100. After setting n_estimators, a second grid search was performed, with 3-fold cross validation, to tune max_depth (values ranging from 1 to 10) and min_samples_split (values ranging from 2 to 102). A third grid search was performed, with 3-fold cross validation, to tune min_samples_leaf (values ranging from 1 to 10). A fourth grid search was performed, with 3-fold cross validation, to tune max_features (values ranging from 1 to 406). A fifth grid search was performed, with 3-fold cross validation, to tune subsample (values ranging from 0.6 to 0.9). A sixth grid search was performed, with 3-fold cross valiation, to tune learning_rate (values ranging from 0.001 to 0.01) and n_estimators (values ranging from 50 to 5000). Finally, after all hyperparameters were selected, 3-fold cross validation was used to calibrate (using isotonic regression) and train the final model. The final tuned model for men had the following hyperparameters: learning_rate = 0.001, n_estimators = 5000, max_depth = 5, min_samples_split = 2, min_samples_leaf = 1, subsample = 0.8, and max_features = 26.

For women, a similar hyperparameter methodology was employed. The final tuned model for women had the following hyperparameters: learning_rate = 0.01, n_estimators = 500, max_depth = 5, min_samples_split = 82, min_samples_leaf = 1, subsample = 0.8, and max_features = 26.

1. GBT: Sequential measurements at three visits

For men, learning_rate was initially fixed at 0.1, min_samples_split fixed at 2, min_samples_leaf fixed at 1, max_depth fixed at 8, max_features set to ‘sqrt’, and subsample fixed at 0.8. A first grid search was performed, with k-fold cross validation in the training set (k=3 folds selected based on runtime considerations), to tune n_estimators with values ranging from 5 to 100. After setting n_estimators, a second grid search was performed, with 3-fold cross validation, to tune max_depth (values ranging from 1 to 10) and min_samples_split (values ranging from 2 to 102). A third grid search was performed, with 3-fold cross validation, to tune min_samples_leaf (values ranging from 1 to 10). A fourth grid search was performed, with 3-fold cross validation, to tune max_features (values ranging from 1 to 406). A fifth grid search was performed, with 3-fold cross validation, to tune subsample (values ranging from 0.6 to 0.9). A sixth grid search was performed, with 3-fold cross valiation, to tune learning_rate (values ranging from 0.001 to 0.01) and n_estimators (values ranging from 50 to 5000). Finally, after all hyperparameters were selected, 3-fold cross validation was used to calibrate (using isotonic regression) and train the final model. The final tuned model for men had the following hyperparameters: learning_rate = 0.001, n_estimators = 5000, max_depth = 3, min_samples_split = 62, min_samples_leaf = 7, subsample = 0.8, and max_features = 26.

For women, a similar hyperparameter methodology was employed. The final tuned model for women had the following hyperparameters: learning_rate = 0.001, n_estimators = 5000, max_depth = 5, min_samples_split = 42, min_samples_leaf = 5, subsample = 0.7, and max_features = 26.

1. GBT: Longitudinal summary of sequential measurements at three visits

For men, learning_rate was initially fixed at 0.1, min_samples_split fixed at 2, min_samples_leaf fixed at 1, max_depth fixed at 8, max_features set to ‘sqrt’, and subsample fixed at 0.8. A first grid search was performed, with k-fold cross validation in the training set (k=3 folds selected based on runtime considerations), to tune n_estimators with values ranging from 5 to 100. After setting n_estimators, a second grid search was performed, with 3-fold cross validation, to tune max_depth (values ranging from 1 to 10) and min_samples_split (values ranging from 2 to 102). A third grid search was performed, with 3-fold cross validation, to tune min_samples_leaf (values ranging from 1 to 10). A fourth grid search was performed, with 3-fold cross validation, to tune max_features (values ranging from 1 to 406). A fifth grid search was performed, with 3-fold cross validation, to tune subsample (values ranging from 0.6 to 0.9). A sixth grid search was performed, with 3-fold cross valiation, to tune learning_rate (values ranging from 0.001 to 0.01) and n_estimators (values ranging from 50 to 5000). Finally, after all hyperparameters were selected, 3-fold cross validation was used to calibrate (using isotonic regression) and train the final model. The final tuned model for men had the following hyperparameters: learning_rate = 0.1, n_estimators = 50, max_depth = 3, min_samples_split = 22, min_samples_leaf = 8, subsample = 0.8, and max_features = 126.

For women, a similar hyperparameter methodology was employed. The final tuned model for women had the following hyperparameters: learning_rate = 0.01, n_estimators = 500, max_depth = 3, min_samples_split = 62, min_samples_leaf = 4, subsample = 0.7, and max_features = 76.

1. GBT: Longitudinal summary of stroke risk estimates at three visits + Single measurement at most recent visit

For men, learning_rate was initially fixed at 0.1, min_samples_split fixed at 2, min_samples_leaf fixed at 1, max_depth fixed at 8, max_features set to ‘sqrt’, and subsample fixed at 0.8. A first grid search was performed, with k-fold cross validation in the training set (k=3 folds selected based on runtime considerations), to tune n_estimators with values ranging from 5 to 100. After setting n_estimators, a second grid search was performed, with 3-fold cross validation, to tune max_depth (values ranging from 1 to 10) and min_samples_split (values ranging from 2 to 102). A third grid search was performed, with 3-fold cross validation, to tune min_samples_leaf (values ranging from 1 to 10). A fourth grid search was performed, with 3-fold cross validation, to tune max_features (values ranging from 1 to 406). A fifth grid search was performed, with 3-fold cross validation, to tune subsample (values ranging from 0.6 to 0.9). A sixth grid search was performed, with 3-fold cross valiation, to tune learning_rate (values ranging from 0.001 to 0.01) and n_estimators (values ranging from 50 to 5000). Finally, after all hyperparameters were selected, 3-fold cross validation was used to calibrate (using isotonic regression) and train the final model. The final tuned model for men had the following hyperparameters: learning_rate = 0.001, n_estimators = 50, max_depth = 3, min_samples_split = 42, min_samples_leaf = 1, subsample = 0.8, and max_features = 41.

For women, a similar hyperparameter methodology was employed. The final tuned model for women had the following hyperparameters: learning_rate = 0.001, n_estimators = 500, max_depth = 3, min_samples_split = 142, min_samples_leaf = 1, subsample = 0.8, and max_features = 71.


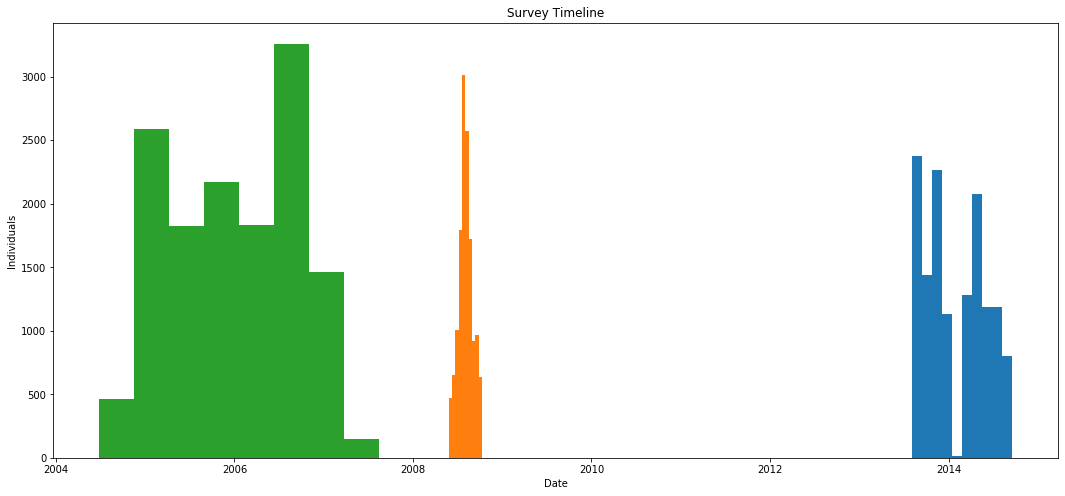


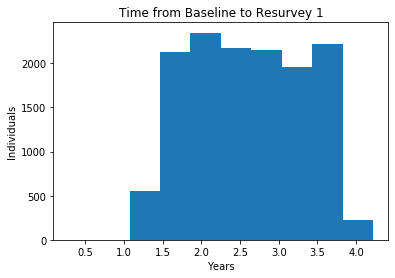

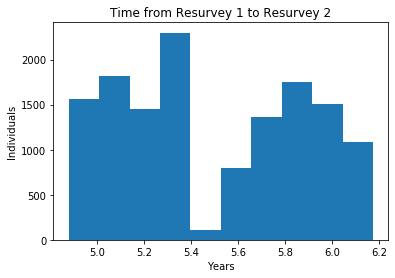


**Supplementary Figure 1.** Timing of baseline questionnaire, resurvey 1, and resurvey 2 for included CKB individuals.


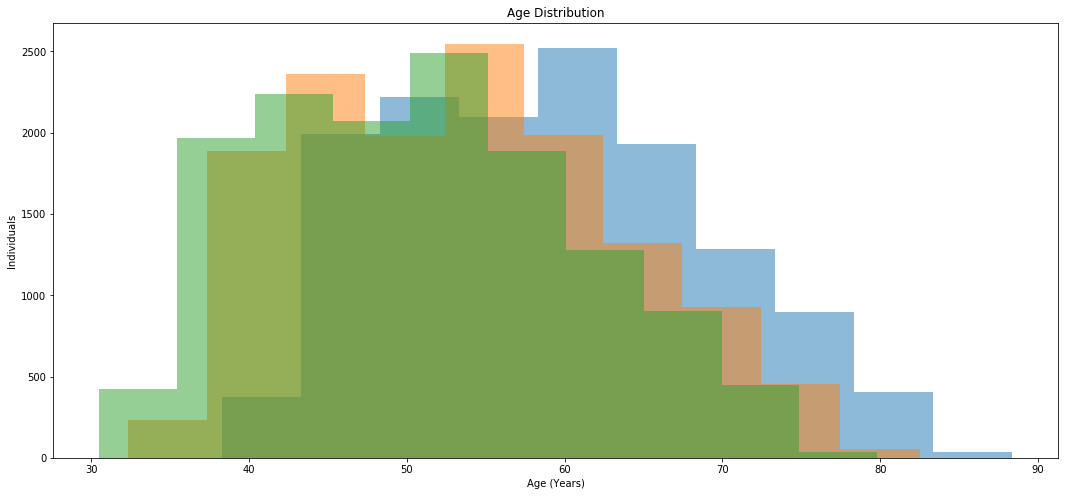


**Supplementary Figure 2.** Distribution of age at baseline (green), resurvey 1 (orange), and resurvey 2 (blue) in included CKB individuals.


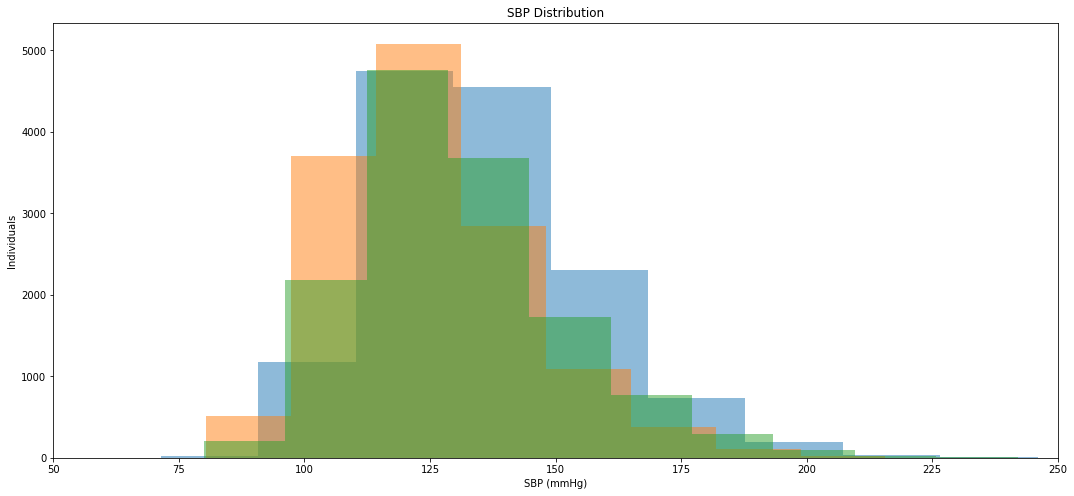


**Supplementary Figure 3.** Distribution of systolic blood pressure at baseline (green), resurvey 1 (orange), and resurvey 2 (blue) in included CKB individuals.

Baseline to Resurvey 1


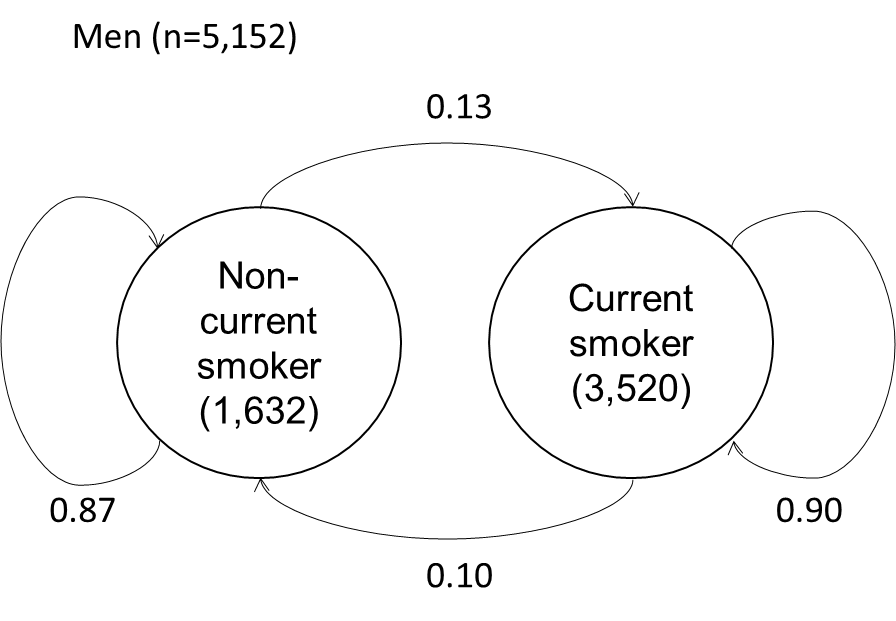

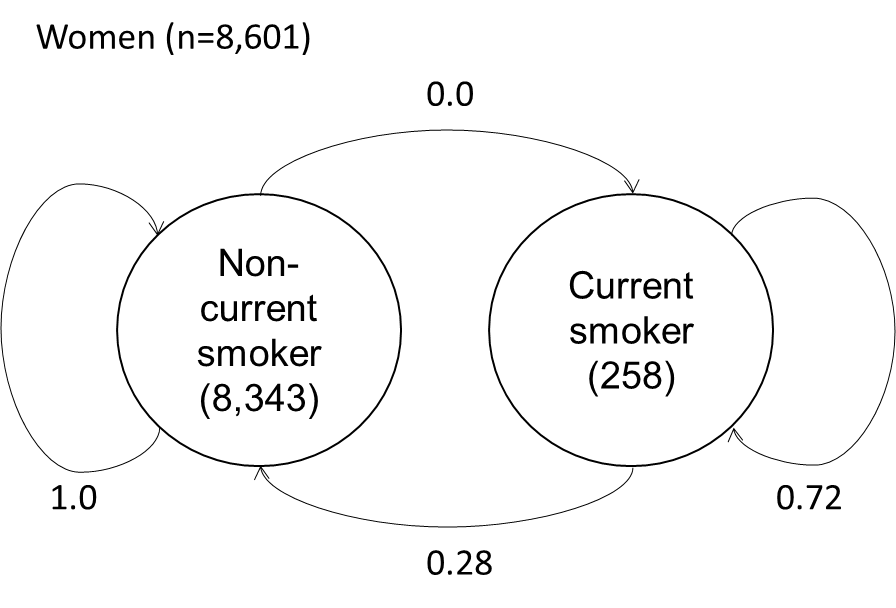


Resurvey 1 to Resurvey 2


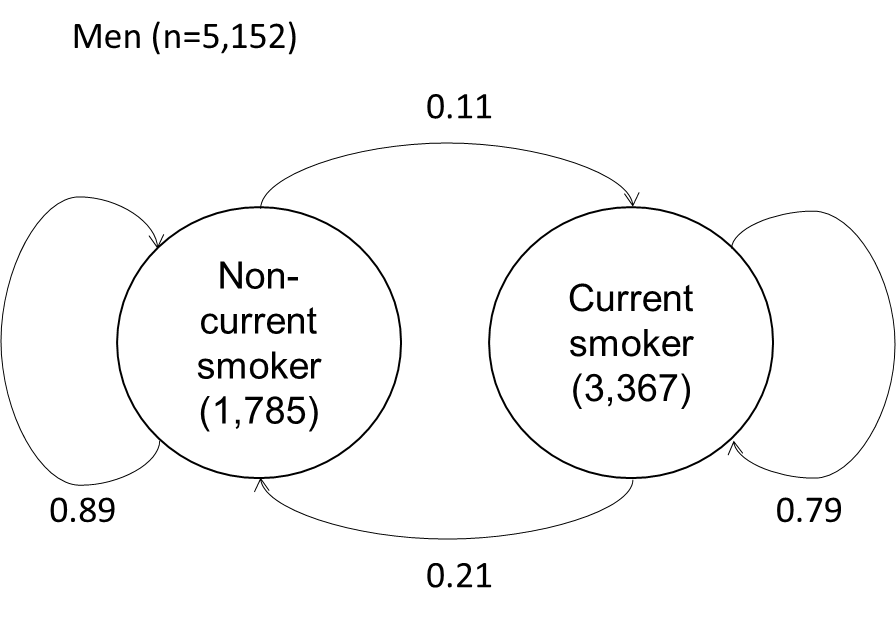

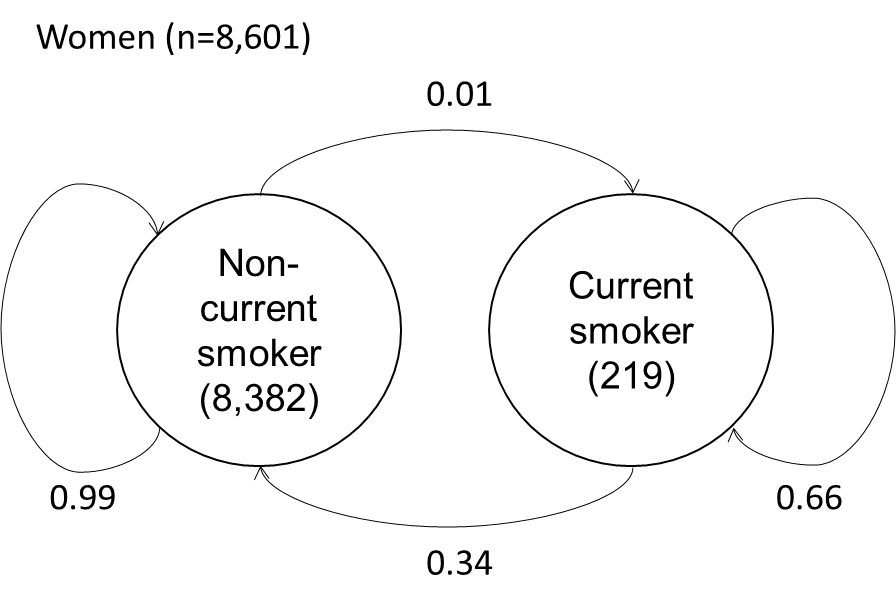


**Supplementary Figure 4.** Distribution of self-reported smoking status and state transitions between baseline, resurvey 1, and resurvey 2 among included CKB individuals.

Baseline to Resurvey 1


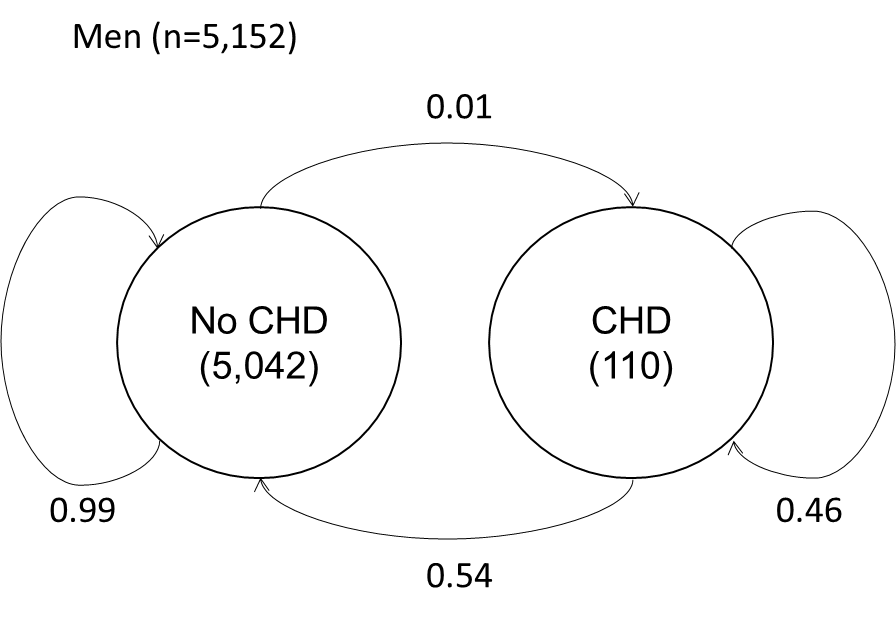

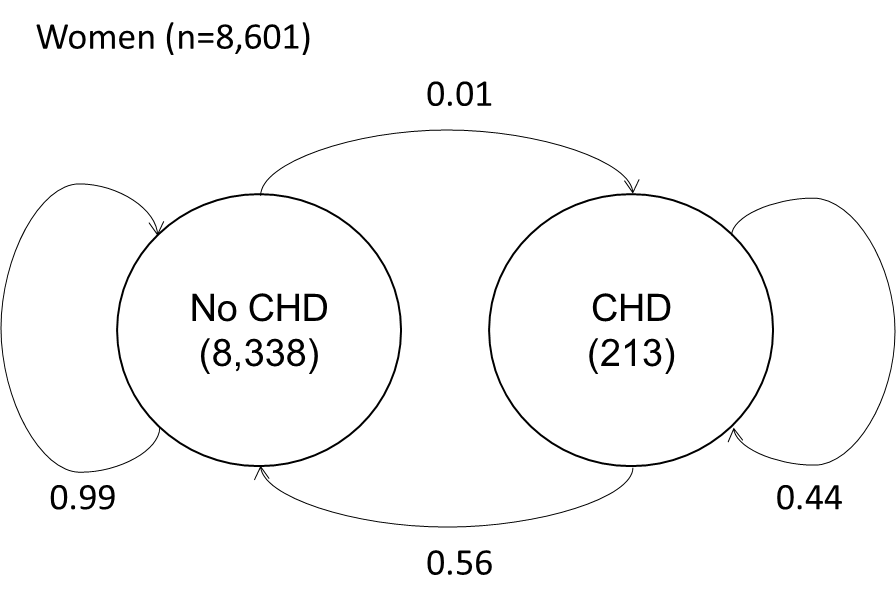


Resurvey 1 to Resurvey 2


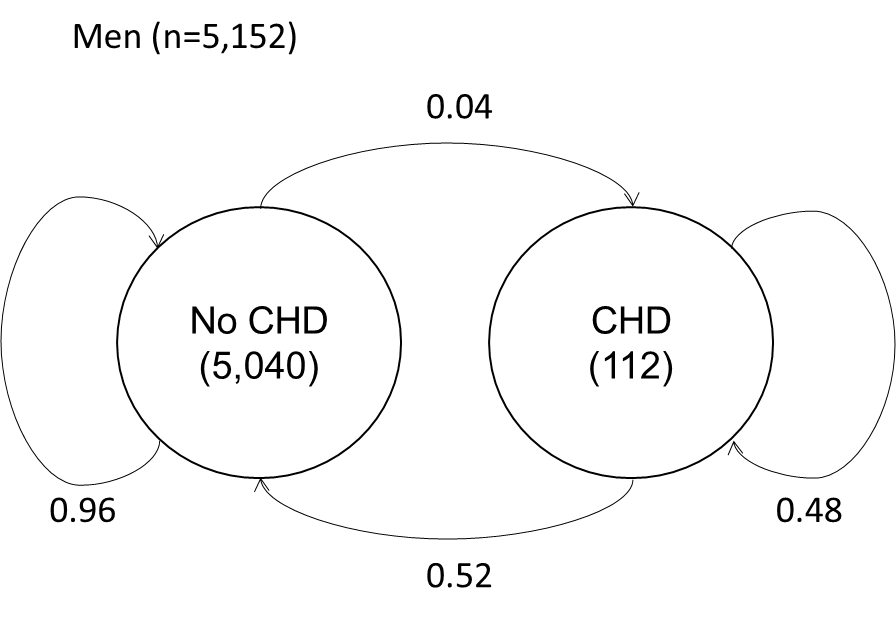

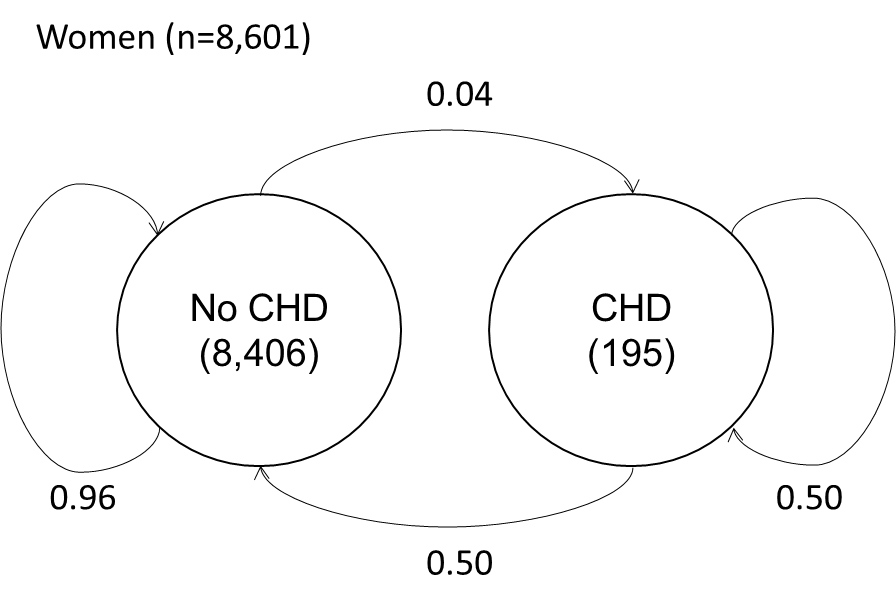


**Supplementary Figure 5.** Distribution of self-reported history of coronary heart disease (CHD) and state transitions between baseline, resurvey 1, and resurvey 2 among included CKB individuals.

Note: No individuals who self-report a history of a CHD diagnosis should properly report that they have no history of a CHD diagnosis at a subsequent visit. To adjust for these self-reporting errors, in our analyses, we considered an individual to have a history of coronary heart disease if they ever reported CHD at a previous visit.

Baseline to Resurvey 1


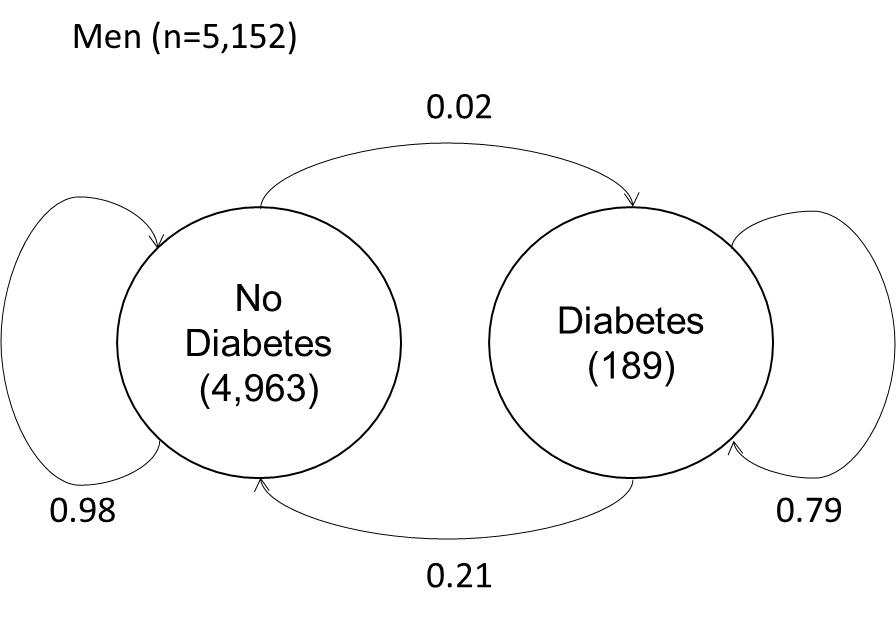

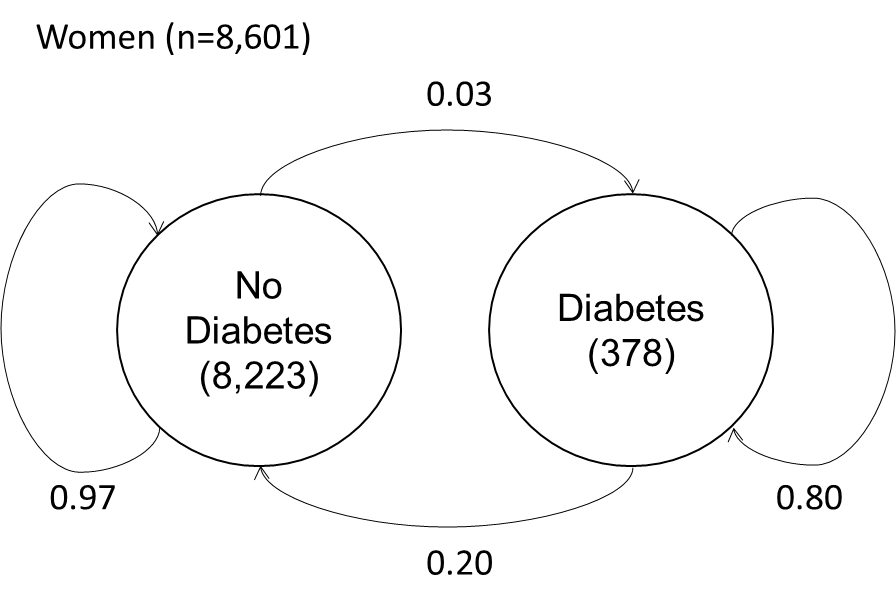


Resurvey 1 to Resurvey 2


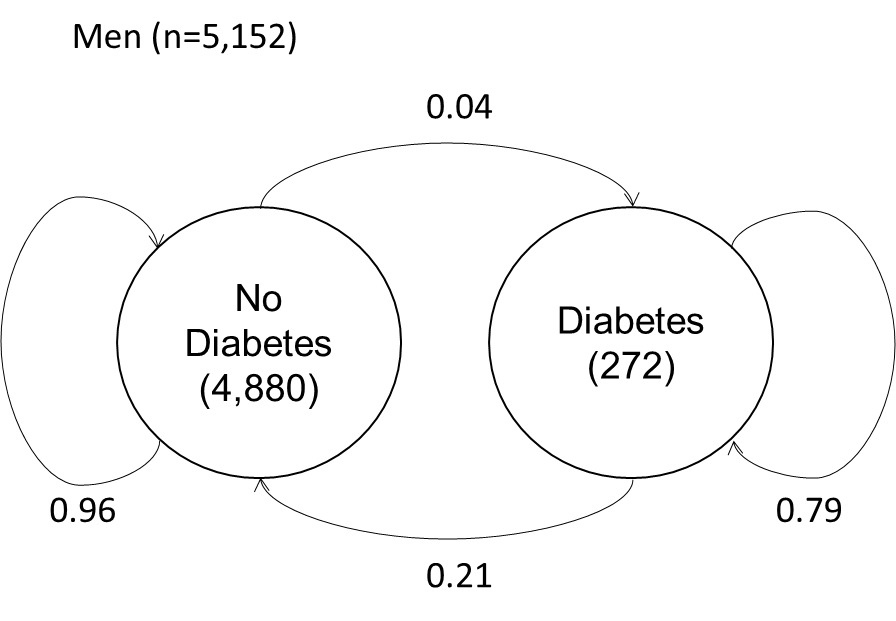

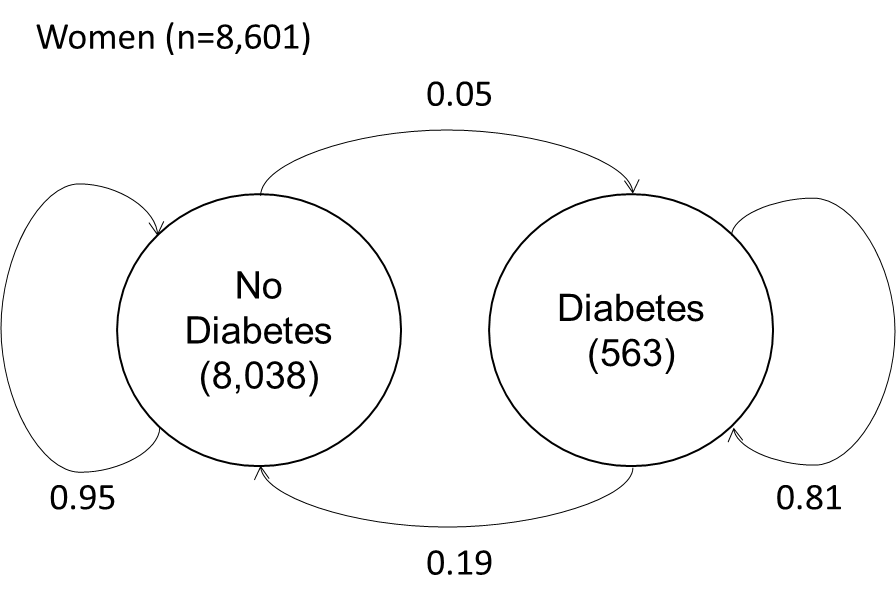


**Supplementary Figure 6.** Distribution of diabetes status and state transitions between baseline, resurvey 1, and resurvey 2 among included CKB individuals. At each survey, diabetes status was recorded based on a combination of self-reporting and detection using blood glucose tests.

Note: No individuals who self-report a history of a diabetes diagnosis or have detected diabetes should properly report that they have no history of a diabetes diagnosis at a subsequent visit. To adjust for these self-reporting errors, in our analyses, we considered an individual to have a history of diabetes if they ever had self-reported or detected diabetes at a previous visit.

Baseline to Resurvey 1


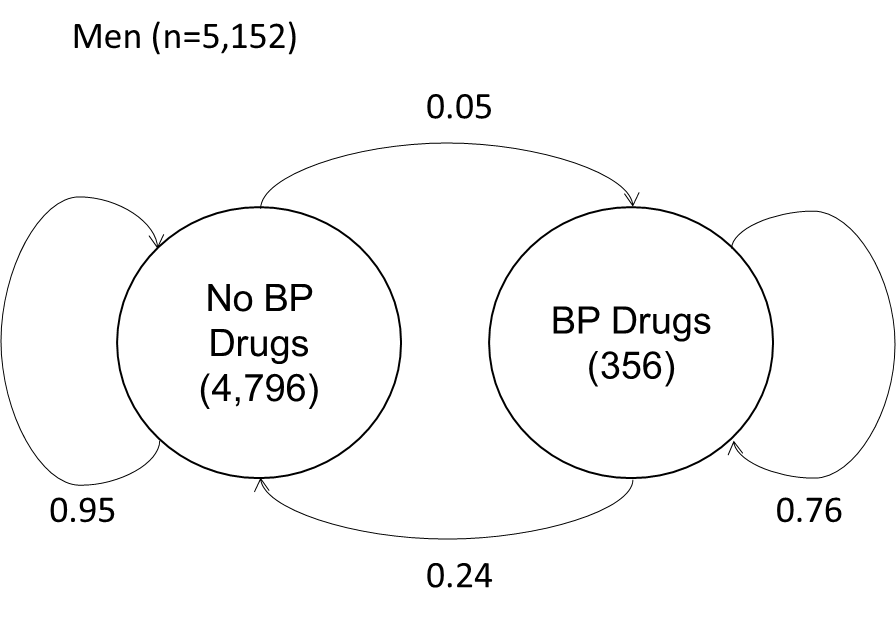

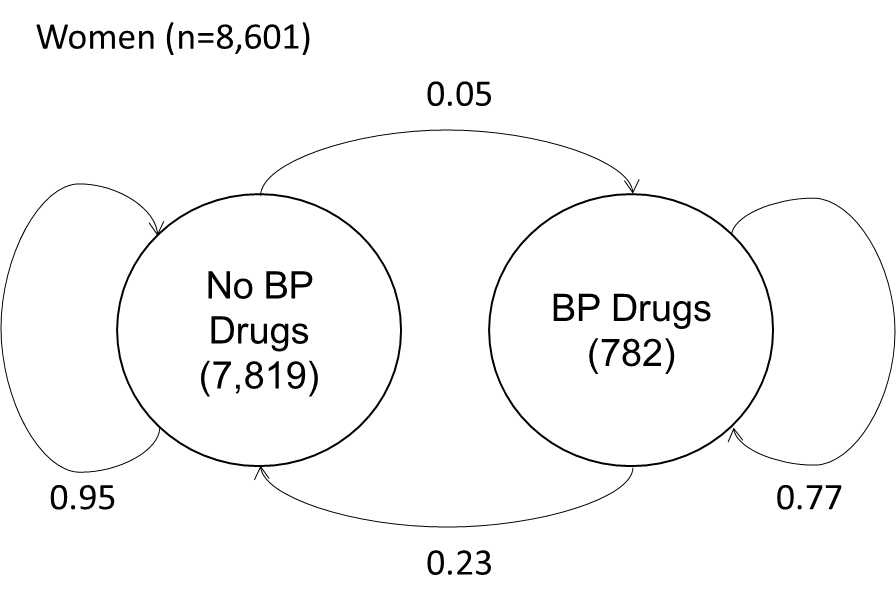


Resurvey 1 to Resurvey 2


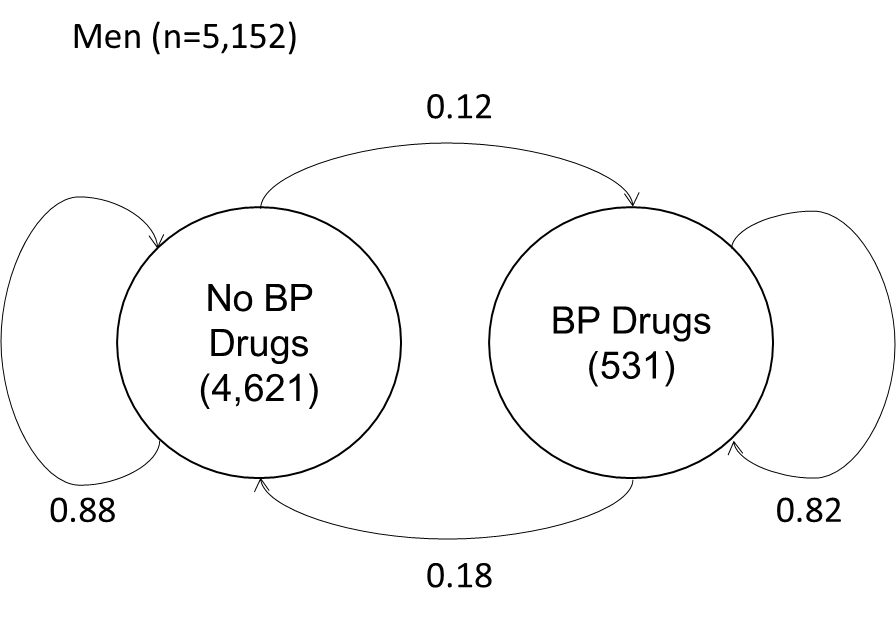
 **
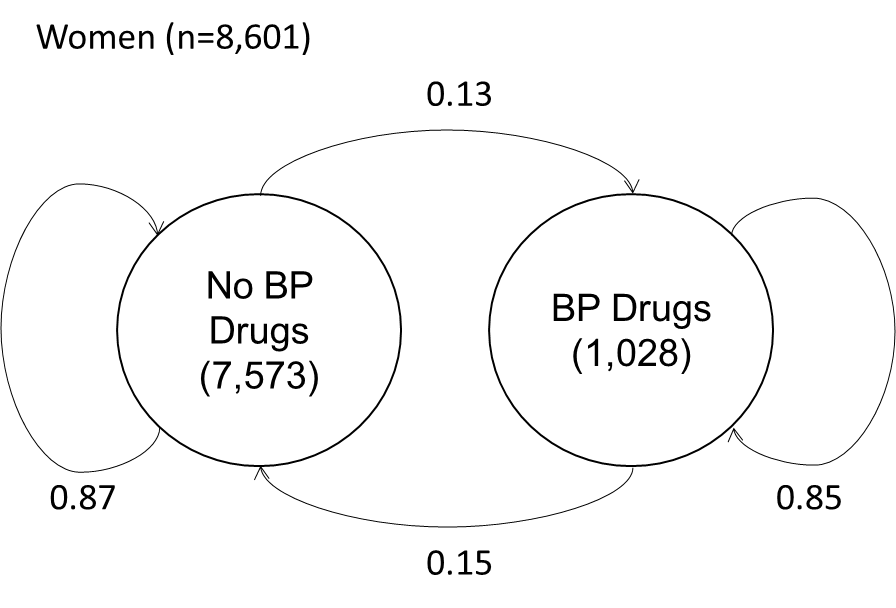
**

**Supplementary Figure 7.** Distribution of self-reported use of antihypertensive medication and state transitions between baseline, resurvey 1, and resurvey 2 among included CKB individuals.


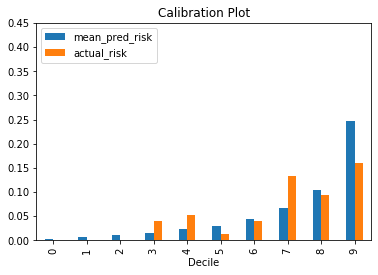

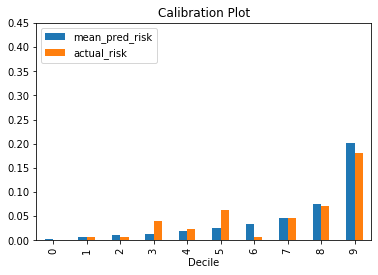
**Supplementary Figure 8.** Calibration plots for “Cox: Single measurement at most recent visit” models in men (left) and women (right).

**
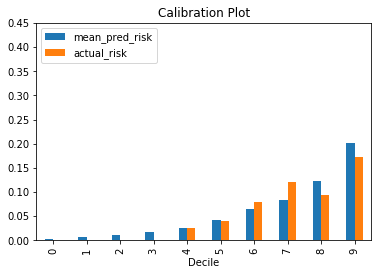

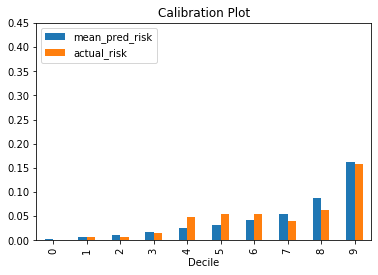
Supplementary Figure 9.** Calibration plots for “GBT: Single measurement at most recent visit” models in men (left) and women (right).

**
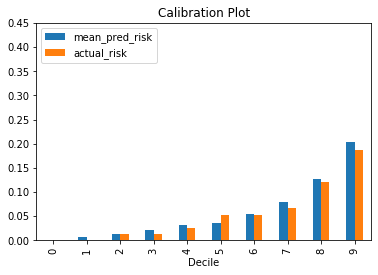

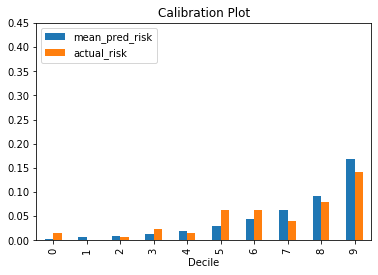
Supplementary Figure 10.** Calibration plots for “GBT: Sequential measurements at three visits” models in men (left) and women (right).

**
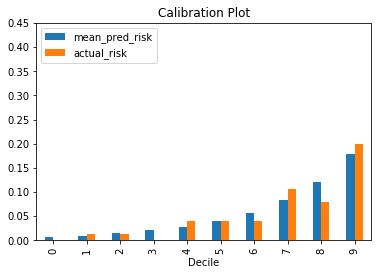

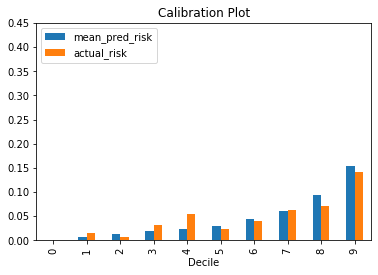
**

**Supplementary Figure 11.** Calibration plots for “GBT: Longitudinal summary of sequential measurements at three visits” models in men (left) and women (right).

**
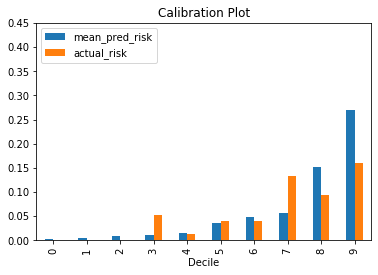

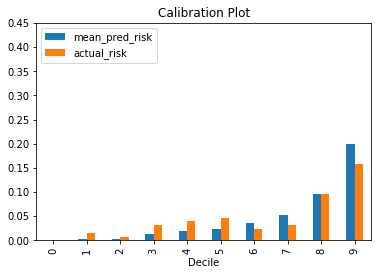
**

**Supplementary Figure 12.** Calibration plots for “Longitudinal summary of stroke risk estimates at three visits + Single measurement at most recent visit” models in men (left) and women (right).

**Supplementary Table 1.** List of all CKB risk factor inputs considered in the present study. All risk factor inputs were recorded at three separate visits, except for sex and region (including urban/rural classification), which were assumed to be unchanged throughout the study. Sex was not included as a risk factor input to the models, since separate models were developed for men and women.

|  |  | **Selected Using LASSO Regularization in:** | |
| --- | --- | --- | --- |
| **Name** | **Definition/Corresponding CKB Survey Question** | **Cox Model (Men)** | **Cox Model (Women)** |
| ***Established Risk Factor Inputs Included in 2017 Framingham Stroke Risk Profile*** | | | |
| Age (10 years) | Age in decades | X | X |
| Current Smoking | Do you currently smoke? (0: no, 1: yes) |  |  |
| Coronary heart disease (CHD) | Has a doctor EVER told you that you had coronary heart disease? (0: no, 1:  yes) | X | X |
| Age 65+ | Age ≥ 65 years? (0: no, 1: yes) |  |  |
| DM, if Age <65 | IF age < 65 years, do you have diabetes? (0: no, 1: yes) | X | X |
| DM, if Age 65+ | IF age < 65 years, do you have diabetes? (0: no, 1: yes) |  |  |
| HTN Rx | Do you use blood pressure drugs? (0: no, 1: yes) | X | X |
| SBP per 10 mmHg, if no HRx | IF not using blood pressure drugs, what is your systolic blood pressure (in  mmHg) / 10 | X | X |
| SBP per 10 mmHg, if HRx | IF using blood pressure drugs, what is your systolic blood pressure (in mmHg) / 10 |  |  |
| ***Geographic Risk Factor Inputs*** | | | |
| region | CKB survey site region (Gansu, Haikou, Harbin, Henan, Hunan, Liuzhou, Qingdao, Sichuan, Suzhou, or Zhejiang) | X | X |
| region_is_urban | Is the region urban? (0: no, 1: yes) | X | X |
| ***Physical Measurements*** | | | |
| sbp_mean | Systolic blood pressure in mmHg (mean of two measurements) | X | X |
| dbp_mean | Diastolic BP in mmHg (mean of two measurements) | X | X |
| heart_rate_mean_10s | Heart rate (beats per minute/10) |  | X |
| has_diabetes | Participant has history of diabetes (reported OR random blood glucose ≥ 11.1  mmol/L OR fasting blood glucose ≥ 7.0 mmol/L) (0: no, 1: yes) | X | X |
| standing_height_cm | Standing height in cm (without shoes) | X | X |
| sitting_height_cm | Sitting height in cm |  |  |
| waist_cm | Waist measurement in cm | X | X |
| waist_hip_ratio_percent | Waist to hip ratio * 100 |  |  |
| weight_kg | Weight in kg (without shoes, but in light clothing) |  |  |
| bmi_calc | BMI calculated from measured height and weight (in kg/m^2^) |  |  |
| fat_percent | Body fat percentage | X |  |
| ***Medical History*** | | | |
| hypertension_diag | Has a doctor EVER told you that you had hypertension? (0: no, 1: yes) | X | X |
| has_copd | Has a doctor EVER told you that you had COPD? (0: no, 1: yes) | X | X |
| rheum_heart_dis_diag | Has a doctor EVER told you that you had rheumatic heart disease? (0: no, 1:  yes) |  | X |
| tb_diag | Has a doctor EVER told you that you had TB? (0: no, 1: yes) | X | X |
| cirrhosis_hep_diag | Has a doctor EVER told you that you had cirrhosis/chronic hepatitis? (0: no, 1: yes) |  | X |
| peptic_ulcer_diag | Has a doctor EVER told you that you had a peptic ulcer? (0: no, 1: yes) |  |  |
| gall_diag | Has a doctor EVER told you that you had a gallstone/gallbladder dis.? (0: no, 1: yes) |  | X |
| asthma_diag | Has a doctor EVER told you that you had asthma? (0: no, 1: yes) |  |  |
| kidney_dis_diag | Has a doctor EVER told you that you had kidney disease? (0: no, 1: yes) | X | X |
| fracture_diag | Has a doctor EVER told you that you had a fracture? (0: no, 1: yes) |  | X |
| rheum_arthritis_diag | Has a doctor EVER told you that you had rheumatoid arthritis? (0: no, 1: yes) |  | X |
| neurasthenia_diag | Has a doctor EVER told you that you had neurasthenia? (0: no, 1: yes) |  | X |
| head_injury_diag | Has a doctor EVER told you that you had a head injury? (0: no, 1: yes) |  | X |
| cancer_diag | Has a doctor EVER told you that you had cancer? (0: no, 1: yes) |  | X |
| blood_transfusions | How many blood transfusions have you received? (if none, put 0) |  |  |
| emph_bronc_diag | Has a doctor EVER told you that you had emphysema/bronchitis? (0: no, 1:  yes) | X |  |
| psych_disorder_diag | Has a doctor EVER told you that you have a psychiatric disorder? (0: no, 1:  yes) |  |  |
| ***Family Medical History*** | | | |
| children | How many children do you have? | X | X |
| siblings | How many siblings do you have? |  | X |
| mother_still_alive | Is your mother still alive? (0: no, 1: yes) | X | X |
| father_still_alive | Is your father still alive? (0: no, 1: yes) | X | X |
| mother_stroke | Did your mother ever have a stroke? (0: no, 1: yes) | X | X |
| mother_heart_attack | Did your mother ever have a heart attack? (0: no, 1: yes) | X |  |
| mother_diabetes | Did your mother ever have diabetes? (0: no, 1: yes) |  |  |
| mother_cancer | Did your mother ever have cancer? (0: no, 1: yes) |  |  |
| father_stroke | Did your father ever have a stroke? (0: no, 1: yes) | X | X |
| father_heart_attack | Did your father ever have a heart attack? (0: no, 1: yes) |  |  |
| father_diabetes | Did your father ever have diabetes? (0: no, 1: yes) |  |  |
| father_cancer | Did your father ever have cancer? (0: no, 1: yes) | X |  |
| siblings_stroke | Did your siblings (incl. half siblings) ever have a stroke? (0: if no, else record the number with disease) | X | X |
| siblings_heart_attack | Did your siblings (incl. half siblings) ever have a heart attack? (0: if no, else record the number with disease) | X | X |
| siblings_diabetes | Did your siblings (incl. half siblings) ever have diabetes? (0: if no, else record the number with disease) |  | X |
| siblings_cancer | Did your siblings (incl. half siblings) ever have cancer? (0: if no, else record  the number with disease) |  |  |
| children_stroke | Did your children ever have a stroke? (0: if no, else record the number with  disease) |  | X |
| children_heart_attack | Did your children ever have a heart attack? (0: if no, else record the number with disease) |  |  |
| children_diabetes | Did your children ever have diabetes? (0: if no, else record the number with  disease) | X | X |
| children_cancer | Did your children ever have cancer? (0: if no, else record the number with  disease) |  |  |
| missing_mother_history | Missing reported history of stroke, heart attack, diabetes, and/or cancer in  mother |  |  |
| missing_father_history | Missing reported history of stroke, heart attack, diabetes, and/or cancer in  father |  |  |
| missing_siblings_history | Missing reported history of stroke, heart attack, diabetes, and/or cancer in  siblings |  |  |
| ***Lifestyle Risk Factor Inputs*** | | | |
| met | Total daily physical activity (Metabolic Equivalent of Task [MET hours/day]) | X | X |
| met_hours | Total daily hours spent on physical activity | X |  |
| smoking_category_1 | Does the following characterise your smoking behaviour? – Never smoker (0: no, 1: yes) |  | X |
| smoking_category_2 | Does the following characterise your smoking behaviour? – Occasional smoker (0: no, 1: yes) |  |  |
| smoking_category_3 | Does the following characterise your smoking behaviour? – Ex regular smoker (0: no, 1: yes) |  | X |
| smoking_category_4 | Does the following characterise your smoking behaviour? – Regular smoker (0: no, 1: yes) | X |  |
| smoking_now_1 | Does the following characterise your smoking behaviour? – Only occasionally (0: no; 1: yes) |  |  |
| smoking_now_2 | Does the following characterise your smoking behaviour? – Yes, on most days (0: no; 1: yes) |  |  |
| smoking_now_3 | Does the following characterise your smoking behaviour? – Yes, daily or almost every day (0: no; 1: yes) | X | X |
| years_since_quitting_smoking | How many years since quitting smoking? (0 if you currently smoke or never smoked; decimal values allowed) | X |  |
| alcohol_category_1 | Does the following characterise your alcohol intake? – Never regular (0: no; 1: yes) | X | X |
| alcohol_category_2 | Does the following characterise your alcohol intake? – Ex-regular (0: no; 1:  yes) | X |  |
| alcohol_category_3 | Does the following characterise your alcohol intake? – Occasional (0: no; 1: yes) | X |  |
| alcohol_category_4 | Does the following characterise your alcohol intake? – Monthly (0: no; 1: yes) |  | X |
| alcohol_category_5 | Does the following characterise your alcohol intake? – Reduced intake (0: no; 1: yes) |  |  |
| alcohol_category_6 | Does the following characterise your alcohol intake? – Weekly (0: no; 1: yes) |  |  |
| diet_freq_rice_0 | During the past 12 months, about how often did you eat rice? – Daily (0: no,  1: yes) |  |  |
| diet_freq_rice_1 | During the past 12 months, about how often did you eat rice? – 4-6 days per  week (0: no, 1: yes) | X |  |
| diet_freq_rice_2 | During the past 12 months, about how often did you eat rice? – 1-3 days per  week (0: no, 1: yes) | X | X |
| diet_freq_rice_3 | During the past 12 months, about how often did you eat rice? – Monthly (0:  no, 1: yes) |  |  |
| diet_freq_rice_4 | During the past 12 months, about how often did you eat rice? – Never/rarely  (0: no, 1: yes) |  |  |
| diet_freq_wheat_0 | During the past 12 months, about how often did you eat wheat? – Daily (0: no,  1: yes) | X | X |
| diet_freq_wheat_1 | During the past 12 months, about how often did you eat wheat? – 4-6 days per  week (0: no, 1: yes) |  | X |
| diet_freq_wheat_2 | During the past 12 months, about how often did you eat wheat? – 1-3 days per  week (0: no, 1: yes) |  |  |
| diet_freq_wheat_3 | During the past 12 months, about how often did you eat wheat? – Monthly (0:  no, 1: yes) | X | X |
| diet_freq_wheat_4 | During the past 12 months, about how often did you eat wheat? – Never/rarely  (0: no, 1: yes) | X | X |
| diet_freq_other_staple_0 | During the past 12 months, about how often did you eat staple foods other  than rice and wheat (corn, millet etc.)? – Daily (0: no, 1: yes) | X | X |
| diet_freq_other_staple_1 | During the past 12 months, about how often did you eat staple foods other  than rice and wheat (corn, millet etc.)? – 4-6 days per week (0: no, 1: yes) | X | X |
| diet_freq_other_staple_2 | During the past 12 months, about how often did you eat staple foods other  than rice and wheat (corn, millet etc.)? – 1-3 days per week (0: no, 1: yes) | X | X |
| diet_freq_other_staple_3 | During the past 12 months, about how often did you eat staple foods other  than rice and wheat (corn, millet etc.)? – Monthly (0: no, 1: yes) |  | X |
| diet_freq_other_staple_4 | During the past 12 months, about how often did you eat staple foods other  than rice and wheat (corn, millet etc.)? – Never/rarely (0: no, 1: yes) | X |  |
| bowel_movement_freq_0 | About how often do you have bowel movements each week? – More than once on most days (0: no, 1:yes) |  |  |
| bowel_movement_freq_1 | About how often do you have bowel movements each week? – About daily (0: no, 1:yes) |  |  |
| bowel_movement_freq_2 | About how often do you have bowel movements each week? – Once every 2-3 days (0: no, 1:yes) |  |  |
| bowel_movement_freq_3 | About how often do you have bowel movements each week? – Less than 3  times a week (0: no, 1:yes) | X |  |
| gum_bleed_freq_0 | How often do your gums bleed when you brush your teeth? – Occasionally,  rarely or never (0: no, 1:yes) |  |  |
| gum_bleed_freq_1 | How often do your gums bleed when you brush your teeth? – Sometimes (0:  no, 1:yes) | X | X |
| gum_bleed_freq_2 | How often do your gums bleed when you brush your teeth? – Always (0: no,  1:yes) |  |  |
| gum_bleed_freq_3 | How often do your gums bleed when you brush your teeth? – Brush teeth  rarely or never (0: no, 1:yes) | X | X |
| ***Socioeconomic Demographic Risk Factor Inputs*** | | | |
| household_size | How many people live together in your household? | X | X |
| has_health_cover | Do you have health care coverage? (0: no, 1: yes) |  |  |
| highest_education_0 | What is the highest level of school education you ever received? – No formal school (0: no, 1: yes) | X | X |
| highest_education_1 | What is the highest level of school education you ever received? – Primary school (0: no, 1: yes) | X | X |
| highest_education_2 | What is the highest level of school education you ever received? – Middle  school (0: no, 1: yes) |  |  |
| highest_education_3 | What is the highest level of school education you ever received? – High school (0: no, 1: yes) | X | X |
| highest_education_4 | What is the highest level of school education you ever received? – Technical school / college (0: no, 1: yes) | X |  |
| highest_education_5 | What is the highest level of school education you ever received? – Technical school / college (0: no, 1: yes) | X |  |
| occupation_0 | What is your current occupation? – Agriculture and related (0: no, 1: yes) |  |  |
| occupation_1 | What is your current occupation? – Factory worker (0: no, 1: yes) | X | X |
| occupation_2 | What is your current occupation? – Administrator / manager (0: no, 1: yes) |  |  |
| occupation_3 | What is your current occupation? – Professional / technical (0: no, 1: yes) |  |  |
| occupation_4 | What is your current occupation? – Sales and service (0: no, 1: yes) |  |  |
| occupation_5 | What is your current occupation? – Retired (0: no, 1: yes) | X | X |
| occupation_6 | What is your current occupation? – House wife / husband (0: no, 1: yes) | X |  |
| occupation_7 | What is your current occupation? – Self-employed (0: no, 1: yes) |  |  |
| occupation_8 | What is your current occupation? – Unemployed (0: no, 1: yes) |  |  |
| occupation_9 | What is your current occupation? – Other or not stated (0: no, 1: yes) |  | X |
| household_income_0 | What is the total income last year in your household? – <2,500 yuan (0: no, 1: yes) |  |  |
| household_income_1 | What is the total income last year in your household? – 2,500-4,999 yuan (0: no, 1: yes) | X |  |
| household_income_2 | What is the total income last year in your household? – 5,000-9,999 yuan (0: no, 1: yes) |  | X |
| household_income_3 | What is the total income last year in your household? – 10,000-19,999 yuan (0: no, 1: yes) | X | X |
| household_income_4 | What is the total income last year in your household? – 20,000-34,999 yuan (0: no, 1: yes) |  | X |
| household_income_5 | What is the total income last year in your household? – ≥35,000 yuan (0: no, 1: yes) | X | X |
| ***Self-Assessed Health Status*** | | | |
| self_rated_health_0 | How would you rate your current general health status? – Excellent (0: no, 1: yes) |  |  |
| self_rated_health_1 | How would you rate your current general health status? – Good (0: no, 1: yes) | X | X |
| self_rated_health_2 | How would you rate your current general health status? – Fair (0: no, 1: yes) | X |  |
| self_rated_health_3 | How would you rate your current general health status? – Poor (0: no, 1: yes) | X | X |
| comparative_health_0 | How would rate your current general health status compared to someone of  your own age? – Better (0: no, 1: yes) | X | X |
| comparative_health_1 | How would rate your current general health status compared to someone of  your own age? – About the same (0: no, 1: yes) |  |  |
| comparative_health_2 | How would rate your current general health status compared to someone of  your own age? – Worse (0: no, 1: yes) | X | X |
| comparative_health_3 | How would rate your current general health status compared to someone of  your own age? – Don’t know (0: no, 1: yes) | X | X |

|  | GBT: Single measurement at most recent visit | | GBT: Sequential measurements at three visits | | GBT: Longitudinal summary of sequential measurements at three visits | | GBT: Longitudinal summary of stroke risk estimates at three visits + Single measurement at most recent visit | |
| --- | --- | --- | --- | --- | --- | --- | --- | --- |
| Importance Ranking | Feature | Gini Importance | Feature | Gini Importance | Feature | Gini Importance | Feature | Gini Importance |
| 1 | Age @ resurvey 2 | 0.093 | Age @ resurvey 1 | 0.079 | Age @ resurvey 2 | 0.122 | Random intercept from LME | 0.213 |
| 2 | SBP per 10 mmHg, if no HRx @ resurvey 2 | 0.051 | Age @ baseline | 0.075 | max (SBP mean) | 0.036 | Random slope from LME | 0.163 |
| 3 | SBP mean @ resurvey 2 | 0.047 | Age @ resurvey 2 | 0.075 | min (sitting height) | 0.030 | Variance of risk estimates | 0.145 |
| 4 | met @ resurvey 2 | 0.043 | SBP per 10 mmHg, if no HRx @ resurvey 2 | 0.057 | SD (DBP mean) | 0.028 | Cox risk estimate @ resurvey 2 | 0.142 |
| 5 | weight_kg @ resurvey 2 | 0.042 | SBP mean @ resurvey 2 | 0.053 | max (DBP mean) | 0.027 | Change in risk estimate from resurvey 1 to 2 | 0.087 |
| 6 | standing height @ resurvey 2 | 0.039 | DBP mean @ resurvey 2 | 0.028 | mean (SBP mean) | 0.021 | Change in risk estimate from baseline to resurvey 1 | 0.048 |
| 7 | DBP mean @ resurvey 2 | 0.038 | waist_cm @ baseline | 0.025 | mean (children) | 0.021 | Age @ resurvey 2 | 0.028 |
| 8 | sitting height @ resurvey 2 | 0.038 | standing height @ resurvey 2 | 0.025 | Haikou | 0.020 | standing height @ resurvey 2 | 0.018 |
| 9 | BMI @ resurvey 2 | 0.034 | SBP mean @ baseline | 0.024 | mean (met_hours) | 0.020 | SBP mean @ resurvey 2 | 0.016 |
| 10 | Heart rate @ resurvey 2 | 0.033 | DBP mean @ baseline | 0.023 | SD (fat percent) | 0.018 | DBP mean @ resurvey 2 | 0.015 |

**Supplementary Table 2.** Top-10 most important features for GBT models in men.

**Supplementary Table 3.** Top-10 most important features for GBT models in women.

|  | GBT: Single measurement at most recent visit | | GBT: Sequential measurements at three visits | | GBT: Longitudinal summary of sequential measurements at three visits | | GBT: Longitudinal summary of stroke risk estimates at three visits + Single measurement at most recent visit | |
| --- | --- | --- | --- | --- | --- | --- | --- | --- |
| Importance Ranking | Feature | Gini Importance | Feature | Gini Importance | Feature | Gini Importance | Feature | Gini Importance |
| 1 | Age @ resurvey 2 | 0.165 | Age @ baseline | 0.046 | Age @ resurvey 2 | 0.068 | Cox risk estimate @ resurvey 2 | 0.217 |
| 2 | waist_cm @ resurvey 2 | 0.078 | Age @ resurvey 1 | 0.044 | Harbin | 0.043 | Random intercept from LME | 0.216 |
| 3 | DBP mean @ resurvey 2 | 0.069 | Age @ resurvey 2 | 0.042 | mean (children) | 0.028 | Random slope from LME | 0.159 |
| 4 | children @ resurvey 2 | 0.061 | met @ resurvey 1 | 0.037 | mean (met_hours) | 0.027 | Variance of risk estimates | 0.135 |
| 5 | fat_percent @ resurvey 2 | 0.059 | Harbin | 0.030 | min (children) | 0.025 | Change in risk estimate from resurvey 1 to 2 | 0.109 |
| 6 | SBP per 10 mmHg, if no HRx @ resurvey 2 | 0.057 | SBP per 10 mmHg, if no HRx @ resurvey 2 | 0.029 | mean (DBP mean) | 0.020 | Change in risk estimate from baseline to resurvey 1 | 0.019 |
| 7 | SBP mean @ resurvey 2 | 0.055 | waist_cm @ resurvey 2 | 0.028 | mean (SBP mean) | 0.020 | fat_percent @ resurvey 2 | 0.017 |
| 8 | standing height @ resurvey 2 | 0.050 | waist_cm @ resurvey 1 | 0.026 | mean (SBP per 10 mmHg, if no HRx) | 0.020 | Age @ resurvey 2 | 0.014 |
| 9 | met_hours @ resurvey 2 | 0.042 | DBP mean @ resurvey 2 | 0.025 | max (waist_cm) | 0.019 | waist_cm @ resurvey 2 | 0.012 |
| 10 | Harbin | 0.042 | DBP mean @ baseline | 0.023 | mean (met) | 0.019 | sitting height @ resurvey 2 | 0.009 |

**References**

1. Chen Z, Chen J, Collins R, et al. China Kadoorie Biobank of 0·5 million people: survey methods, baseline characteristics and long-term follow-up. *Int J Epidemiol*. 2011;40:1652-66.
2. Chen Y, Wright N, Guo Y, et al. Mortality and recurrent vascular events after first incident stroke: a 9-year community-based study of 0.5 million Chinese adults. *Lancet Glob Health*. 2020;8:e580-90.
3. Breslow NE. Discussion of the paper by D.R. Cox. *J R Statist Soc B.* 1972;34:215-16.
4. Lin DY. On the Breslow estimator. *Lifetime Data Anal.* 2007;13:471-80.
5. Friedman J, Hastie T, Tibshirani R. Glmnet: lasso and elastic-net regularized generalized linear models. R package version 3.0-2. 2019. http://CRAN.R-project.org/package=glmnet. Accessed April 8, 2020.
6. Hastie T, Qian J. Glmnet vignette. 2014. http://www.web.stanford.edu/~hastie/Papers/Glmnet_Vignette.pdf. Accessed April 8, 2020.
7. Bates D, Mächler M, Bolker B, Walker S. Fitting Linear Mixed-Effects Models Using lme4. *Journal of Statistical Software*. 2015;67,1–48.
8. Pedregosa F, Varoquaux G, Gramfort A, et al. Scikit-learn: machine learning in Python. *JMLR*. 2011;12:2825-30.
